# Validity and Sensitivity of an Inertial Measurement Unit-Driven Biomechanical Model of Motor Variability for Gait

**DOI:** 10.3390/s21227690

**Published:** 2021-11-19

**Authors:** Christopher A. Bailey, Thomas K. Uchida, Julie Nantel, Ryan B. Graham

**Affiliations:** 1 School of Human Kinetics, University of Ottawa, Ottawa, ON K1N 6N5, Canada; cbailey2@uottawa.ca (C.A.B.); jnantel@uottawa.ca (J.N.); 2 Department of Mechanical Engineering, University of Ottawa, Ottawa, ON K1N 6N5, Canada; thomas.uchida@uottawa.ca

**Keywords:** gait, inertial measurement unit, joint kinematics, local dynamic stability, OpenSim, persistence, regularity, stride-to-stride variability

## Abstract

Motor variability in gait is frequently linked to fall risk, yet field-based biomechanical joint evaluations are scarce. We evaluated the validity and sensitivity of an inertial measurement unit (IMU)-driven biomechanical model of joint angle variability for gait. Fourteen healthy young adults completed seven-minute trials of treadmill gait at several speeds and arm swing amplitudes. Trunk, pelvis, and lower-limb joint kinematics were estimated by IMU- and optoelectronic-based models using OpenSim. We calculated range of motion (ROM), magnitude of variability (meanSD), local dynamic stability (λ_max_), persistence of ROM fluctuations (DFAα), and regularity (SaEn) of each angle over 200 continuous strides, and evaluated model accuracy (RMSD: root mean square difference), consistency (ICC_2,1_: intraclass correlation), biases, limits of agreement, and sensitivity to within-participant gait responses (effects of speed and swing). RMSDs of joint angles were 1.7–9.2° (pooled mean of 4.8°), excluding ankle inversion. ICCs were mostly good to excellent in the primary plane of motion for ROM and in all planes for meanSD and λ_max_, but were poor to moderate for DFAα and SaEn. Modelled speed and swing responses for ROM, meanSD, and λ_max_ were similar. Results suggest that the IMU-driven model is valid and sensitive for field-based assessments of joint angle time series, ROM in the primary plane of motion, magnitude of variability, and local dynamic stability.

## 1. Introduction

Possessing too much or too little motor variability, the natural variability in sensorimotor actions [1], is well linked to walking-related fall risk. These actions include whole-body and joint motions that vary over time from stride to stride. Elderly fallers exhibit greater stride-to-stride variability in spatiotemporal outputs (e.g., stride time) compared to non-fallers [2,3]. This difference may emerge from altered stride-to-stride joint angle patterns that have been observed with older age, including lower local dynamic stability (measured by the local divergence exponent) [4], lower regularity (measured by the sample entropy) [5], and a shift in the magnitude of variability in ankle motion from the sagittal to the frontal plane (measured by the standard deviation) [6]. For example, calculation of phase-dependent entropy of trunk motion during walking was recently shown to improve prediction of future single-time fallers in community-dwelling older adults [7]. Monitoring the variability of joint angles in aging adults at a larger scale could help better understand the salient elements of stride-to-stride control that predict falls, which could better individualize fall-prevention interventions. However, measurements of joint angles (and variability from stride to stride) typically rely on optoelectronic motion capture systems that are expensive, require training and expertise to operate, and involve intensive data acquisition and processing procedures. Optoelectronic motion capture of gait is also restricted to treadmills [4,5] or short distances overground [6], which may not fully replicate the stride-to-stride variability that occurs over longer distances and durations overground. Using this motion capture approach, large-scale evaluations of motor variability in realistic and clinically-relevant gait scenarios are infeasible.

Wearable inertial measurement units (IMUs) offer an alternative technology for estimating joint angles that can address these limitations of optoelectronic motion capture. IMUs have been used to estimate joint kinematics since 1990 [8], with recent work showing that IMU-based kinematic models of lower-limb activities achieve absolute differences (i.e., accuracies) ranging from 1 to 10° relative to optoelectronic-based models [9,10,11,12,13,14,15] and good–excellent consistencies in the sagittal plane time series [9,12,16,17]. Some IMU-based kinematic models are built based on machine learning approaches [13], but most others typically involve (i) estimating the IMU orientation by fusing accelerometer and gyroscope data, (ii) estimating the anatomical segment orientation by applying a sensor-to-segment calibration (e.g., [18]), and (iii) calculating joint angles as the relative orientation between reference frames fixed to adjacent body segments (see [19] for review). A particular obstacle to the longer-duration recordings necessary to measure stride-to-stride kinematics is preventing IMU sensor orientation drift attributed to sensor fusion. Strap-down integration of an IMU on a non-stabilized segment amplifies random noise in linear accelerations and angular velocities, leading to drift in the estimated orientations. Many sensor fusion algorithms address horizontal drift by incorporating data on the Earth’s magnetic field detected by the magnetometer [20,21,22,23,24]; however, detection of the magnetic field is disturbed locally by ferromagnetic materials [25]. Beyond the magnetometer, drift corrections are possible over short durations by adding zero-velocity updates [26] or over short distances by adding localization using technologies like ultrawideband [11], but these approaches do not provide a solution for long durations and distances. 

Anatomical joint constraints in the underlying kinematic model can help to mitigate drift over long durations and distances. Using the OpenSense toolkit to compute inverse kinematics of a biomechanical model with IMU inputs, Al Borno et al. [15] recently demonstrated root mean squared differences (RMSD) of 3–6° for IMU-based hip flexion, hip abduction, knee flexion, and ankle dorsiflexion angles relative to optoelectronic motion capture, with near-zero drift (from 0.14 to 0.17°/min) over ten minutes of walking. This supports the earlier findings of Kok et al. [27] and provides a new open-source option for constructing an IMU-based kinematic model. This inverse kinematics approach to solving joint angles has the added benefit of mitigating experimental (i.e., non-biological) noise [28], which could particularly benefit evaluations of motor variability. Although constrained optimization problems like inverse kinematics can require high computation time to solve, recent work from Slade et al. [14] demonstrated that a real-time IMU-based solution is possible. They reported RMSD in joint angles of approximately 5°, a difference accepted as reasonable for many clinical applications [29]. The OpenSense extension of OpenSim [30,31] provides the first open-source platform for IMU-based biomechanical modelling and a free alternative to cost-prohibitive and closed-source commercial models [20]. Because Al Borno et al. [15] used the magnetometer to calculate drift-free kinematics and Slade et al. [14] reported kinematic drifts in their magnetometer-free solution, it remains unclear whether a magnetometer-free, IMU-based biomechanical model can provide accurate joint angles during gait beyond one minute duration. Furthermore, it is unknown whether IMU-based biomechanical models are valid for evaluating stride-to-stride variability or are sufficiently sensitive to changes in gait kinematics for evaluating fall risk.

The goal of this study was to validate a magnetometer-free, open-source, IMU-based biomechanical model of joint angles and stride-to-stride variability for a moderate duration of continuous gait. We determined the concurrent validity of IMU-based and optoelectronic-based model measurements of joint angles and joint angle variability from the trunk down using OpenSim, and determined the sensitivity of discrete measurements of joint angles and joint angle variability to different gait conditions. We hypothesized that joint angle and joint angle variability measurements from the IMU-based model would be accurate and consistent with the optoelectronic-based model, and that changes in angles and angle variability under different gait conditions would be detected similarly in the two models.

## 2. Methods

### 2.1. Participants

Fourteen healthy young adults (7 males, 7 females) were recruited as a convenience sample from the Ottawa, Canada area. While the IMU-based model is intended for use in older adults as well, young adults were tested to first establish feasibility of the methods and modelling. This sample size (*n* = 12, with 2 extra to account for possible data attrition) was determined a priori using G*Power [32] based on our sensitivity analyses and reflects the number of participants needed to detect a large effect size (partial η^2^ ≥ 0.14) for a within-group factor with three measurement levels (speed: preferred, slow, fast; swing: preferred, active, bound) at a power of 0.80 and an α of 0.05. Participants were excluded if they had a musculoskeletal injury in the preceding 6 months, or any chronic neurological or orthopaedic disorders. Participants all provided written informed consent to this study, which followed the Declaration of Helsinki and was approved by the University of Ottawa Research Ethics Board (H-01-21-6261). 

### 2.2. Instrumentation

**Figure 1:**
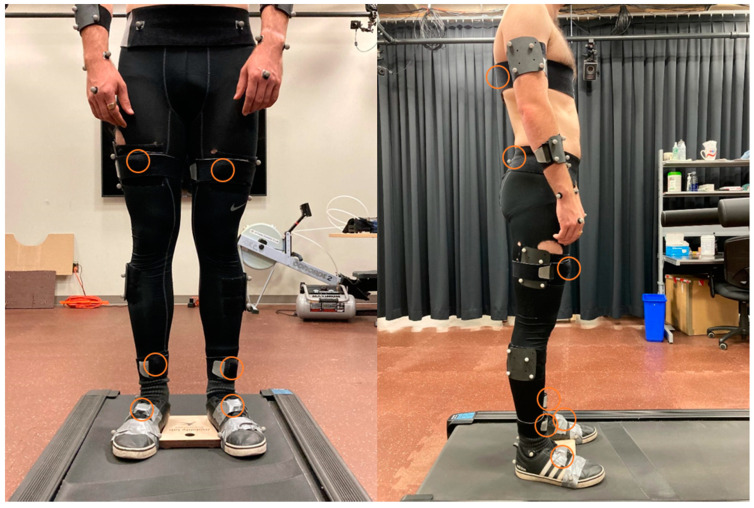
Participant in standing pose on the treadmill showing the dynamic marker set, inertial measurement unit positions (strapped to segments, circled in orange), and the wooden block.

Following informed consent, the participants donned spandex motion capture pants and their own athletic shoes. Participants were then instrumented for optoelectronic- and IMU-based motion capture (Figure 1). An 11-camera optoelectronic system (Vantage, Vicon, Oxford, UK) sampled trajectories of spherical retroreflective markers at 120 Hz using Nexus 2.11 (Vicon, Oxford, UK). Markers were placed on the participant’s body using double-sided tape, following the marker locations used with the full-body model for gait simulations in OpenSim from Rajagopal et al. (Link: https://simtk.org/projects/full_body (accessed on 1 March 2021)) [33]. Anatomical markers were placed on each wrist (radial and ulnar styloid process), on each elbow (medial and lateral epicondyle), on the trunk (left and right acromion process, right clavicular head, spinous process of C7), on the pelvis (left and right anterior superior iliac spine, left and right posterior superior iliac spine), on each knee (medial and lateral femoral condyle), on each ankle (medial and lateral malleolus), and on each foot (heel, 1st and 5th metatarsal head). The dynamic marker set was modified to add redundancy such that rigid-body clusters of four markers (rather than three) were positioned using Velcro straps on the trunk and each forearm, arm, thigh, and shank. Anatomical markers on the feet, pelvis, and the lateral malleoli doubled as dynamic markers for these segments.

IMU-based motion capture was performed using a platform of eight wearable sensors (Dot, Xsens, Enschede, The Netherlands) and a mobile application (Xsens Dot Precision Motion Tracking) for synchronized acquisition of the raw accelerometer and gyroscope data from each sensor. IMU sensors were positioned on the feet (top of the shoe), shanks (anterior aspect, distal and immediately above the malleoli), thighs (anterior aspect, around the largest circumference), pelvis (posterior aspect under the posterior superior iliac spines), and trunk (posterior aspect at the level of the sternum). IMUs were oriented such that the positive xyz axes in the sensor frame in anatomical position were directed leftward, upward, and forward, respectively. Raw accelerations and angular velocities were sampled at 60 Hz using the mobile application in data logging mode. 

### 2.3. Experimental Procedure

Following instrumentation, preferred gait speed was identified according to the procedure of Dingwell and Marin [34]. With the participant blinded to the speed and walking slowly on the treadmill, gait speed was progressively increased until they reported that the speed was “faster than preferred”. Speed was then progressively decreased until they reported that the speed was “slower than preferred”. This sequence was then repeated three times, with the average of the six speeds defined as the preferred gait speed. 

Following a static calibration of the anatomical markers with the participant in standing pose, the participant completed five gait trials on the treadmill (Horizon Fitness, Cottage Grove, WI, USA). Each trial began with a 30 s procedure to warm up the sensors, with the feet oriented on the treadmill using a wooden block to minimize inter-trial and inter-individual differences in foot excursion posture (Figure 1). The participant stood quietly for the first 5 s, leaned forward for the next 10 s, then returned to quiet standing for the final 15 s. After this baseline procedure, the block was removed and the participant completed seven minutes of walking. This sequence was repeated for five trials, each under a different gait condition that varied by gait speed and/or arm swing magnitude: (1) preferred gait speed and arm swing; (2) 70% preferred gait speed, preferred arm swing; (3) 130% preferred gait speed, preferred arm swing; (4) preferred gait speed, active arm swing (the participant was instructed to swing their arms such that forward swing peaked when the arm was horizontal); and (5) preferred gait speed, arms bound to the torso (using straps across the arms and across the elbows). These gait conditions with different speeds and arm swing amplitudes were evaluated since they have been shown to alter stride-to-stride variability patterns in gait [35,36,37,38,39], providing a basis for exploring the sensitivity of the IMU-based model. Gait conditions each lasted seven minutes to record at least six minutes of consecutive and constant-speed strides. This duration was needed to reach a minimum of 150 consecutive strides for stable measurements of motor variability [40] and is also the duration of the six-minute walk test [41], a common assessment of functional mobility. Condition order was randomized, the participant rested for a minimum of three minutes between trials, and optoelectronic and IMU data were continuously sampled during each trial.

### 2.4. Data Analysis

#### 2.4.1. Optoelectronic-Based Biomechanical Modelling

Marker trajectories were labelled, gap-filled with a Woltring spline [42], and low-pass filtered at 10 Hz using Nexus (v2.11, Vicon Inc., Oxford, UK). Filtered trajectories were then imported into OpenSim v4.1 [31] and used to simulate motion of a full-body model containing 37 degrees of freedom (DOF) and 80 muscle–tendon units actuating the lower limbs [33]. This model includes a 3-DOF trunk (relative to pelvis; flexion, lateral bending, rotation), a 6-DOF pelvis (relative to ground; tilt, obliquity, rotation), 3-DOF hips (flexion, abduction, rotation), 1-DOF knees (flexion), 2-DOF ankles (flexion, inversion), and 1-DOF metatarsophalangeal joints (flexion). For simplicity and consistency, angles in the sagittal, frontal, and transverse planes will be described as flexion/extension (FE), abduction/adduction (AA), and internal/external rotation (IE), respectively. The 1-DOF toe joints were locked since toe motion was not recorded by IMUs. The model was scaled to the participant using the positions of the anatomical markers in the static trial; joint angles in each trial were then computed via an inverse kinematic analysis that minimized the least-squared distance between each pair of experimental and model markers. Upper-limb markers were not included in the analysis since no IMUs were placed on the upper limbs and applying weights to the upper-limb markers that equaled weights of lower-limb markers did not influence the inverse kinematics solution (Supplementary Material: Figure S1). Marker weights were manually selected to minimize the root-mean-square error over all marker pairs, resulting in equal weights except for weights of twice the magnitude for markers on the acromion processes (trunk), anterior and posterior superior iliac spines (pelvis), and lateral malleoli (shanks).

#### 2.4.2. IMU-Based Biomechanical Modelling

Using Matlab (R2020b, The MathWorks Inc., Natick, MA, USA), raw linear accelerations and angular velocities were fused offline to calculate sensor orientations using the magnetometer-free algorithm of Madgwick et al. [21]. Orientation drifts were then removed using a detrending procedure. Beginning at the 20 s timestamp (5 s after the participant had completed the forward lean and was standing quietly) and endingj at trial completion, quaternions were converted to ZYX Euler angles and fit to a function using ‘polyfit’. Linear drift was identified as slope less than −0.0010 rad/s or greater than 0.0010 rad/s and removed from the Euler angle signal using ‘detrend’, then Euler angles were converted back to quaternion representation. An example showing the orientation of a sensor before and after drift removal is provided in the Supplementary Material (Figure S2). 

Detrended sensor quaternions were imported into OpenSim using the OpenSense toolkit to simulate motion of the same full-body biomechanical model [33]. Sensor-to-segment registration was performed to associate the orientation of each sensor with the corresponding segment in the model; specifically, each thigh, shank, and foot sensor was registered, respectively, to each femur, tibia, and talus body segment. Sensor orientations were converted from their local coordinate systems to the OpenSim coordinate system using the following sequence of body-fixed rotations: 180° about x, then 90° about y, and finally −90° about z. IMU segment frames were identified based on the standing pose at the start of each gait trial: fixed rotational offsets were applied to recorded IMU sensor frames based on the segment frames of the biomechanical model in a neutral standing pose (i.e., joint flexion of 0°), with heading offsets applied to individual IMU sensor frames to match the average heading and align with the anterior–posterior axis of the biomechanical model [14,15]. As with the optoelectronic-based model, joint angles in each trial were calculated via inverse kinematics; for the IMU-based model, the solver minimized axis-angle differences between the IMU segment orientations and IMU sensor orientations [15]. We compensated for differences between the initial pose of the optoelectronic and IMU models by offsetting optoelectronic-based joint angle time series by a constant to match the neutral standing pose of the IMU model. In contrast with the single ankle dorsiflexion/plantarflexion DOF modelled previously [14,15], we chose to also model ankle inversion/eversion since both sagittal- and frontal-plane ankle compensations are relevant to gait of aging adults [6]. We explored optoelectronic- and IMU-based inverse kinematic solutions of the 1-DOF and 2-DOF ankle models and confirmed that the additional ankle DOF did not affect the inverse kinematics solutions for other lower-limb joint angles (Supplementary Material: Figure S1). Example animations of the optoelectronic- and IMU-based biomechanical models can be viewed in the Supplementary Material (Videos S1–S4).

### 2.5. Calculation of Kinematic Outcomes

Time series of optoelectronic- and IMU-modelled joint angles and linear velocities of the foot segments were exported to Matlab. Strides were partitioned by the first and subsequent heel strike events, identified from the tri-dimensional linear velocity vectors of the calcaneus segments. Linear velocities were low-pass Butterworth filtered with zero lag (10 Hz cutoff, fourth-order) and the Euclidean norm linear velocity vector was then calculated. From the Euclidean norm linear velocity, heel strike events were identified as the local minima immediately following each local maximum (Supplementary Material: Figure S3). After removing strides in the first 30 s to ensure gait was at steady state, the raw and time-normalized series (101 points per stride: 0–100%) of joint angles were analyzed for the subsequent 200 strides. (This was the minimum number of synchronized strides available for all trials and participants.) Five outcomes were then calculated for each joint angle, as described below.

#### 2.5.1. Range of Motion (ROM)

The difference between the maximum and minimum angles for each stride in the time-normalized series. The mean value across strides was computed.

#### 2.5.2. Mean Standard Deviation (meanSD)

A measure of the absolute magnitude of variability, meanSD was calculated using the time-normalized series. SD was calculated across all strides for each time point (*n* = 101 points) and the mean SD across time points (meanSD) was computed.

#### 2.5.3. Maximum Finite-Time Lyapunov Exponent (λ_max_)

A measure of local dynamic stability, λ_max_ (i.e., the local divergence exponent) was calculated using the continuous series. The continuous series was normalized to 20,000 points (100 per stride on average), then λ_max_ was computed with 5 embedding dimensions at a lag of 10 points from 0–0.5 strides (50 points) [4,43]. λ_max_ measures the local divergence of neighbouring trajectories with higher positive values indicative of higher divergence and lower local dynamic stability.

#### 2.5.4. Detrended Fluctuation Analysis Scaling Exponent (DFAα)

A measure of statistical persistence, DFAα was calculated as the fluctuation in ROM across strides, computed as previously described [44,45] and quantifying the extent to which ROM fluctuations statistically persist. DFAα is non-negative and unitless, with values greater than 0.5 indicating persistence (i.e., a fluctuation is typically followed by a fluctuation in the same direction), values less than 0.5 indicating anti-persistence (i.e., a fluctuation is typically followed by a fluctuation in the opposite direction), and values around 0.5 indicating no correlation between consecutive fluctuations [46]. 

#### 2.5.5. Sample Entropy (SaEn)

A measure of signal regularity, SaEn was calculated using the continuous series, 2 embedding dimensions, and a 0.15 tolerance distance [47]. SaEn can be investigated at several scales using a multiscale function; we selected a scale factor of 4 which is believed to be the approximate value where entropy of physiological signals stabilizes during self-selected slow, normal (usual), and fast walking speeds [47]. To compensate for the influence of sampling frequency [48], IMU-based joint angles were resampled at 120 Hz to match the optoelectronic system. SaEn is non-negative and unitless, with higher values indicating lower regularity.

### 2.6. Statistical Analyses

#### 2.6.1. Analyses of IMU-Model Validity

Statistical analyses were completed using SPSS (v27, IBM, Armonk, NY, USA). For each degree of freedom, we assessed concurrent validity of the IMU-based joint angles and angle outputs relative to the optoelectronic-based joint angles and angle outputs for 1000 strides (5 gait conditions × 200 strides). Mean RMSD was calculated for the time-normalized joint angle series as well as for each outcome variable. For the time series analysis, relative difference was calculated as the coefficient of variation of RMSD relative to the optoelectronic-based ROM (CV_rom_); for the outcome variable analysis, relative difference was calculated relative to the optoelectronic-based mean (CV_mean_). Intraclass correlation coefficients (ICC_2,1_) and Bland–Altman plot metrics (IMU–optoelectronic measurement bias, 95% limits of agreement) were computed to examine consistency and agreement between outcomes. ICC_2,1_ values less than 0.40, from 0.40 to 0.59, from 0.60 to 0.74, and greater than or equal to 0.75 were interpreted as poor, fair, good, and excellent consistency, respectively [49]. Biases indicated whether IMU-based outcomes were overestimated (positive bias) or underestimated (negative bias) on average.

#### 2.6.2. Analyses of IMU-Model Sensitivity

For each degree of freedom and outcome, we assessed the sensitivity of the IMU model to detect the same within-participant changes as the marker-based model by conducting repeated measures ANOVAs on each model to test for effects of gait speed (speed: preferred, 70% preferred, 130% preferred) and arm swing (swing: preferred, active, bound). Greenhouse–Geisser corrections were applied if sphericity was violated, and critical alpha was set to 0.027 using the Benjamini–Hochberg procedure to account for false discovery rate due to multiple comparisons [50] (240 *p*-values: 2 models × 2 statistical effects × 12 angles × 5 outcomes). Post hoc tests, comparing each condition to preferred speed and preferred arm swing gait, were made with Bonferroni corrections (*p* < 0.05).

## 3. Results

### 3.1. Participant Characteristics

Participant height, mass, and BMI (mean ± SD) averaged 1.72 ± 0.07 m, 69.6 ± 14.2 kg, and 23.5 ± 3.9 kg/m^2^, respectively. Gait at preferred speed averaged 1.12 ± 0.18 m/s (range: 0.72–1.50), at 70% preferred speed averaged 0.79 ± 0.13 m/s (range: 0.50–1.05 m/s), and at 130% preferred speed averaged 1.46 ± 0.24 m/s (range: 0.93–1.95).

### 3.2. IMU-Model Validity

#### 3.2.1. Validity of Joint Angle Time Series

**Figure 2:**
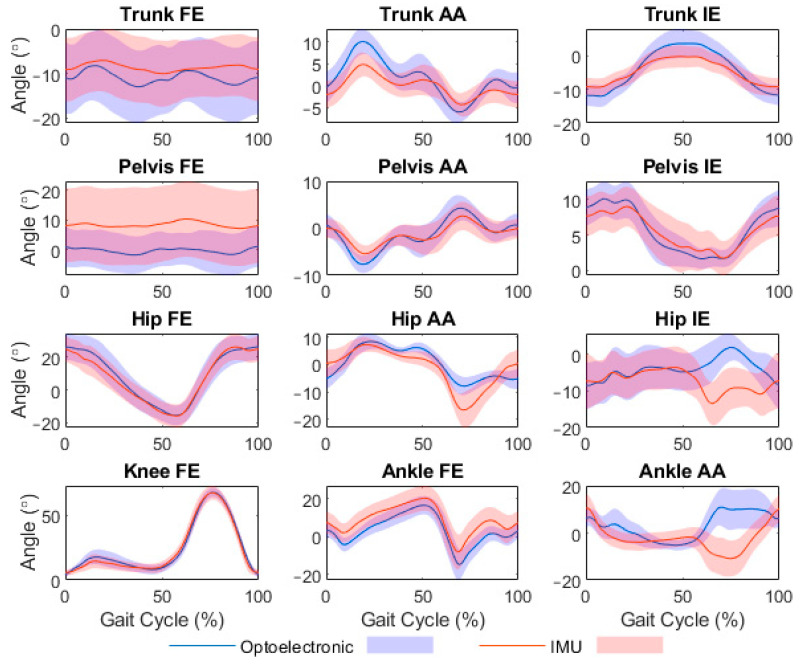
Ensemble averaged joint angles from the optoelectronic and inertial measurement unit (IMU) models during gait at preferred speed and with preferred arm swing. Angles include flexion/extension (FE), abduction/adduction (AA), and internal/external rotation (IE).

Mean values are presented in Table 1, with ensemble averaged curves for the preferred speed condition displayed in Figure 2. Based on values pooled across conditions, mean RMSD was less than 5° for all trunk angles, pelvis AA and IE, hip FE, and knee FE, with all other angles except pelvis FE and ankle AA approaching the 5° threshold. RMSD pooled across conditions and angles was 5.3°, which dropped to 4.8° when ankle AA was excluded. RMSDs were consistent across 200 consecutive strides, showing that IMU-modelled joint angles did not drift (Supplementary Material: Figure S4). CV_rom_ averaged 26.9% across angles, being lowest in the transverse plane for the trunk (16.2%) and pelvis (12.8%), and lowest in the sagittal plane for the hip (9.2%), knee (6.4%), and ankle (17.1%).

#### 3.2.2. Validity of Joint Angle Range of Motion and Motor Variability Outcomes

Mean values are presented in Table 2, with Bland–Altman plots for each outcome displayed in Supplementary Material (Figures S5–S9). Good–excellent consistency was seen for ROM of trunk IE, pelvis AA, pelvis IE, hip FE, and ankle FE (ICC_2,1_: 0.62–0.85), for meanSD of all angles except knee FE and ankle AA (ICC_2,1_: 0.60–0.80), for λ_max_ of all angles except trunk IE and ankle AA (ICC_2,1_: 0.67–0.89), for DFAα of trunk IE and ankle FE (ICC_2,1_: 0.62–0.65), and for SaEn of trunk AA, pelvis AA, pelvis IE, and knee FE (ICC_2,1_: 0.61–0.74). 

Differences [absolute: RMSD (relative: CV_mean_)] between outcomes with good–excellent consistency were 2.0–7.7° (5.0–38.3%) for ROM, 0.14–0.61° (10.9–50.0%) for meanSD, 0.20–0.61 (3.9–17.8%) for λ_max_, 0.16–0.17 (16.6–17.2%) for DFAα, and 0.07–0.24 (15.1–63.1%) for SaEn. 

Significant mean biases (defined as cases where the 95% confidence interval did not cross zero; Table 2) indicated that the IMU model underestimated ROM of the trunk, pelvis, and ankle FE by 0.4–7.0°, but overestimated ROM of frontal plane hip and ankle angles by 3.1–6.8°. For stride-to-stride variability outcomes, the IMU model underestimated meanSD of trunk IE and pelvis AA by 0.08–0.52°, λ_max_ of trunk IE by 0.23, DFAα of pelvis AA and hip AA by 0.07, and SaEn of pelvis FE, hip AA, hip IE, knee FE, and ankle AA by 0.02–0.12. These outcomes were more frequently overestimated by the IMU model, with higher meanSD (0.04–0.56°) and λ_max_ (0.20–1.13) for most angles, higher DFAα of ankle AA (0.12), and higher SaEn of trunk AA, trunk IE, pelvis AA, pelvis IE, hip FE, and ankle FE (0.04–0.19). These biases approximated the optoelectronic-measured inter-individual standard deviations in our sample and in measurements from other studies for joint angle ROM [51], meanSD [35,39], DFAα [39], and SaEn [5,39], but exceeded inter-individual standard deviations for joint angle λ_max_ [4,39]. The Bland–Altman plots (Supplementary Material: Figures S5–S9) show that nearly all measurement differences (14 participants × 5 conditions = 70 values) fell within the 95% limits of agreement, with few outliers in ROM (N = 1–5), meanSD (N = 2–5), λ_max_ (N = 1–4), DFAα (N = 1–5), and SaEn (N = 0–6) of individual angles.

### 3.3. IMU-Model Sensitivity

Precise descriptives (means, 95% confidence intervals) and statistical effects (*p*-values) for each outcome can be found in Supplementary Material (videos S1 and S2). Since consistency was generally poor to moderate for DFAα and SaEn, indicating a lack of acceptable concurrent validity, the sensitivity of these outcomes was not explored. 

**Figure 3:**
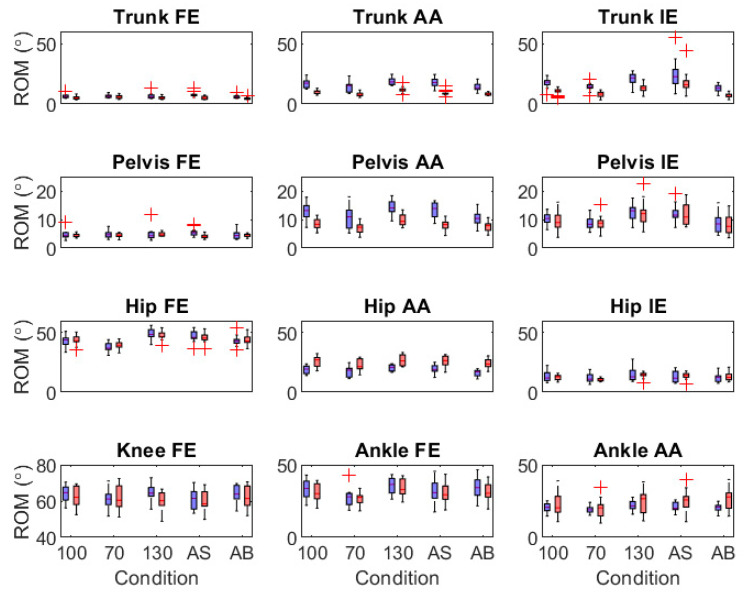
Boxplots of range of motion (ROM) of trunk, pelvis, and lower-limb joint angles for the optoelectronic-driven (blue) and IMU-driven (red) biomechanical models of constant-speed treadmill gait. Conditions displayed on the horizontal axis are preferred speed and arm swing (100), 70% preferred speed (70), 130% preferred speed (130), active arm swing (AS), and arms bound (AB). Red crosses are outliers > 2 * interquartile range. Angles include flexion/extension (FE), abduction/adduction (AA), and internal/external rotation (IE).

**ROM (Figure 3)**. Speed effects: The models detected similar responses relative to preferred-speed gait, with decreased ROM of trunk AA, trunk IE, pelvis AA, hip FE, hip IE, and ankle FE at 70% preferred speed, and increased ROM of trunk AA, trunk IE, pelvis AA, pelvis IE, hip angles, and ankle FE at 130% preferred speed. Differing responses, where only one model detected a significant change, were found at 70% preferred speed for pelvis IE, hip AA, knee FE, and ankle AA, and at 130% preferred speed for ankle AA. The changes detected, however, followed the same trends of decreased ROM at 70% preferred speed and increased ROM at 130% preferred speed. Swing effects: The models also detected similar changes relative to preferred arm swing during active swing, with increased ROM of pelvis IE and hip FE, decreased ROM of knee FE, and no change in ROM of trunk FE, trunk AA, pelvis FE, pelvis AA, hip AA, hip IE, ankle FE, and ankle AA. With arms bound, both models detected decreased ROM of trunk IE and pelvis AA, and no change for pelvis FE, hip FE, hip IE, ankle FE, and ankle AA. 

**Figure 4:**
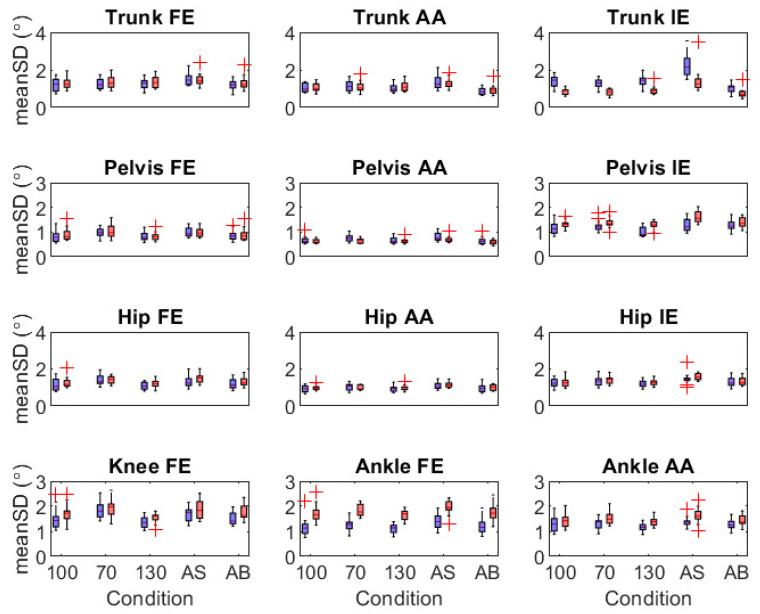
Boxplots of mean standard deviation (meanSD) of trunk, pelvis, and lower-limb joint angles for the optoelectronic-driven (blue) and IMU-driven (red) biomechanical models of constant-speed treadmill gait. Conditions displayed on the horizontal axis are preferred speed and arm swing (100), 70% preferred speed (70), 130% preferred speed (130), active arm swing (AS), and arms bound (AB). Red crosses are outliers > 2 * interquartile range. Angles include flexion/extension (FE), abduction/adduction (AA), and internal/external rotation (IE).

**meanSD (Figure 4)**. Speed effects: The models detected similar non-responses to speed changes for meanSD of trunk FE, trunk IE, ankle FE, and ankle AA. However, optoelectronic-based model increases in meanSD at 70% preferred speed (trunk AA, pelvis FE, pelvis AA, hip FE, hip AA, knee FE) and at 130% preferred speed (pelvis IE) went undetected by the IMU-based model. Swing effects: Relative to preferred arm swing, the models detected similar increases in meanSD (trunk AA, pelvis AA, pelvis IE, hip AA, hip IE) and non-responses (trunk FE, hip FE, knee FE) during active arm swing, as well as similar non-responses with arms bound (trunk FE, pelvis FE, pelvis AA, and all hip, knee, and ankle angles). Optoelectronic-detected increases in meanSD of trunk AA and IE and decreases for pelvis IE with arms bound went undetected by the IMU-based model.

**Figure 5:**
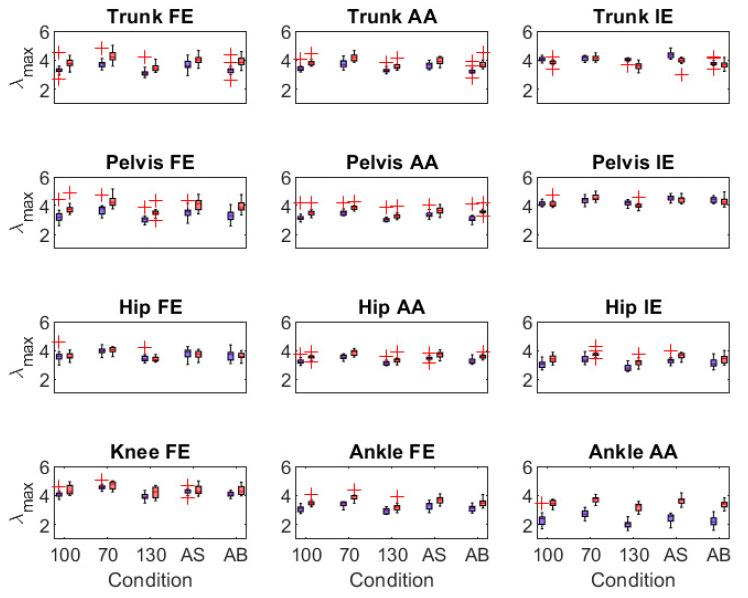
Boxplots of local divergence exponent (λ_max_) of trunk, pelvis, and lower-limb joint angles for the optoelectronic-driven (blue) and IMU-driven (red) biomechanical models of constant-speed treadmill gait. Conditions displayed on the horizontal axis are preferred speed and arm swing (100), 70% preferred speed (70), 130% preferred speed (130), active arm swing (AS), and arms bound (AB). Red crosses are outliers > 2 * interquartile range. Angles include flexion/extension (FE), abduction/adduction (AA), and internal/external rotation (IE).

**λ_max_ (Figure 5)**. Speed effects: The models detected similar responses to speed changes in λ_max_ of joint angles with few exceptions. Both models detected increases in λ_max_ at 70% preferred speed (trunk FE, trunk AA, pelvis-down angles), decreases in λ_max_ at 130% preferred speed (trunk FE, pelvis FE, pelvis AA, hip AA, hip IE, ankle AA), and no response in λ_max_ of knee FE at 130% preferred speed. Swing effects: The models also detected similar λ_max_ responses relative to preferred arm swing, with increases in trunk FE, pelvis angles, hip AA, hip IE, and ankle FE during active swing, and decreases in trunk IE, but increases in pelvis IE, with arms bound. Similar non-responses in λ_max_ of ankle AA during active swing and of trunk FE, hip FE, hip AA, knee FE, ankle FE, and ankle AA with arms bound were also seen.

## 4. Discussion

### 4.1. Validity of IMU-Modelled Joint Angle Time Series

Using a full-body biomechanical model with muscle-actuated lower limbs [33] to analyze several 7-min conditions of gait kinematics, our findings confirm that driving this model with IMUs (using the OpenSense open-source toolkit for OpenSim) produces, on average, accurate joint angle time series relative to the optoelectronic-driven model, with the exception of pelvis tilt, swing-phase hip rotation and swing-phase ankle inversion. RMSD across all gait speed and arm swing conditions averaged 5.3°; when ankle inversion was excluded from this calculation, RMSD averaged 4.8°, an accuracy viewed as acceptable for many clinical applications [29]. This amount of accuracy and the range in RMSD among individual joints (1.7–7.5°) agrees with recent findings from the developers [14,15]. One observation from our ensemble-averaged lower-limb joint angles (Figure 2) was that optoelectronic–IMU differences were notable in the non-sagittal plane during swing (e.g., hip abduction, hip rotation, and ankle inversion), potentially due to the relatively small ROMs of these angles in combination with the higher segmental velocities during swing. Nonetheless, we report an accuracy for hip rotation (pooled RMSD of 6.2°) that improved upon the developers’ findings (10–12°). Because allowing ankle inversion/eversion in addition to dorsiflexion/plantarflexion did not affect the inverse kinematics solution for hip rotation (see, for example, Figure S1), we believe our improvement in accuracy is attributable to omitting magnetometer data when using Madgwick’s gradient-descent fusion algorithm [21] and, instead, identifying negative (<−0.0010 rad/s) and positive (>0.0010 rad/s) linear orientation drifts and detrending these offline. Extending previous findings, we also found RMSDs of IMU-modelled trunk and pelvis angles (1.7–10.6°) that were similar to those of the joints in the lower limb. Thus, with the notable exceptions of pelvis tilt, hip rotation, and ankle inversion, our findings indicate that joint angle time series for the trunk and below can be estimated on average with acceptable accuracy during walking over a wide range of walking speeds and arm swing amplitudes.

### 4.2. Validity of IMU-Modelled Joint Angle Outcomes

The concurrent validity of the joint angle time series, however, did not extend to the discrete outcomes to the same extent. For ROM, as reported by Beange et al. [52,53], optoelectronic–IMU consistency was excellent in the primary plane of movement (trunk rotation, pelvis rotation, hip flexion, ankle dorsiflexion) with RMSDs similar to the corresponding time series (ROM: 2.0–7.7°; time series: 1.9–6.9°), but consistency was often poor in the non-primary movement planes. Therefore, measurement consistency of ROM for the secondary and tertiary planes was not improved by using a biomechanically constrained kinematic model. RMSDs for the secondary and tertiary planes (1.8–8.5°) were comparable to those of the primary movement plane, confirming the findings of Beange et al. [52,53] and likely due to the lower ROMs about the secondary and tertiary planes; future IMU-modelling work of lower-limb joint kinematics could investigate whether consistency is higher in locomotor tasks with similar segmental velocities that include larger ROMs in the frontal and transverse planes, such as walking with side-stepping and/or turning. ROM was also below proposed limits of agreement of ±10° [54] for only 6 of the 12 degrees of freedom we analyzed, with underestimation biases (trunk rotation and ankle flexion) partly responsible. Together, these findings show that acceptable validity of a joint angle time series does not necessarily translate to acceptable validity in the discrete outcomes of that time series. Specifically, our IMU model estimates joint angle ROM accurately, but has inconsistencies in the non-primary movement planes and limitations in absolute agreement.

For motor variability outcomes, consistency was best for λ_max_ and meanSD, reaching good–excellent levels for nearly all degrees of freedom with RMSDs of 0.22–0.61° and 0.14–0.61°, respectively. Our findings agree with the high optoelectronic–IMU consistency in λ_max_ reported for repetitive spine flexion/extension [55] and support the concurrent validity of IMU-based measurements of joint local dynamic stability. For the first time, we demonstrate high consistency and accuracy for joint angles with low range of motion and beyond the primary plane of movement, providing a new validated model for investigating magnitude of variability and local dynamic stability in tridimensional joint motion from stride to stride. Caution is advised regarding the absolute agreement of our outcomes since we were unable to identify limits of agreement in the literature for comparison and since the IMU-based model biases showed that λ_max_ and meanSD were each underestimated for trunk rotation but overestimated for hip, knee, and ankle angles.

DFAα and SaEn were not sufficiently consistent to be considered valid in their present computational form (DFAα ICC_2,1_: −0.05–0.65; SaEn ICC_2,1_: −0.12–0.74). DFAα inconsistencies may be partly derived from ROM inconsistencies since we investigated fluctuations of this discrete metric. This is supported by our finding that the only two angles with good consistency in DFAα (trunk rotation, ankle dorsiflexion) also had excellent consistency in ROM, indicating that improvement in calculation of ROM is likely to produce more valid analyses of fluctuation persistence. SaEn inconsistencies may be due to changes in the information of joint angles in the IMU-driven model. The calculation of this metric for continuous series of joint angles is sensitive to the sampling frequency, the tolerance ratio r, and the embedding dimension m [48]. We resampled IMU-based joint angles to compensate for sampling frequency and selected r and m values which produced stable measurements in slow and fast gait [47]. However, specific tuning of SaEn parameters to IMU-based kinematic models is likely needed. Alternatively, calculation of SaEn may be more robust to tuning parameters by analyzing the discrete ROM series rather than the continuous joint angle series [48], but this also relies on improving the estimation of ROM. Therefore, use of IMU-based biomechanical models to investigate the persistence and regularity of joint angle fluctuations in gait requires improvements to the estimation of ROM and the identification of appropriate SaEn calculation parameters for IMU-derived joint angles. 

### 4.3. Sensitivity of IMU-Modelled Joint Angle Outcomes to Within-Participant Effects

Our results show, for the first time, that joint angle outcomes estimated from an IMU-driven biomechanical model were also sensitive to within-participant responses detected by an optoelectronic-driven model in the majority of cases. Relative to gait at preferred speed and with preferred arm swing, responses to changes in speed and arm swing amplitude were detected identically between models for 36/48 comparisons for ROM, 27/48 comparisons for meanSD, and 34/48 comparisons for λ_max_. Model-detected responses are in agreement with several previous findings, including increases in joint ROM with fast speed and decreases with slow speed [56], increases in SD of trunk kinematics and meanSD of lower-limb angles with active arm swing [38,39], and increases in λ_max_ of hip abduction with active arm swing [39]. Although our trunk motion responses appear to disagree with reported increases in meanSD with fast speed [35], increases in λ_max_ with fast speed [36], and decreases in λ_max_ with active arm swing [37], differences can be attributed to the reference frame of the trunk (relative to the pelvis in our study vs. relative to ground in [35]) and to the sensitivity of the λ_max_ state-space to different inputs [57] (time-delayed joint angles in our study vs. velocities and accelerations [36] vs. time-delayed velocities [37]). In the minority of cases where our optoelectronic and IMU models detected different responses, directionality never disagreed, and differences were mostly due to the IMU-based model detecting no response when a change was detected by the optoelectronic-based model. In these cases, there were no statistically significant changes for the IMU-based model, but all means moved in the same direction as the optoelectronic-based model (Tables S1 and S2). As the probability of this due to chance is very low, it seems that IMU-modelled responses had smaller effect sizes than this study was powered to detect, due to smaller changes in magnitude and/or larger group variance. Furthermore, the excess limits of agreement for some ROMs did not influence sensitivity of the IMU model, as responses in ROM for angles exceeding limits of ±10° [54] were the same in many cases during gait at fast speed (trunk abduction, trunk rotation, ankle dorsiflexion), at slow speed (trunk abduction, trunk rotation, hip abduction, ankle dorsiflexion), with active arm swing (trunk abduction, pelvis flexion, hip abduction, knee flexion, ankle dorsiflexion, ankle inversion), and with bound arms (trunk rotation, ankle flexion, ankle inversion). Together, these findings show that the IMU-driven biomechanical model is sensitive to within-group responses in ROM, meanSD, and λ_max_, and suggest that larger sample sizes may further improve sensitivity by compensating for smaller effect sizes.

### 4.4. Limitations

IMU-driven biomechanical simulations of movement are a new area for investigation and have important limitations. In our study, these were specific to the demographics of our sample, the biomechanical model, and how gait was studied. As a first step for modelling motor variability in gait, we recruited a convenience sample of healthy young adult males and females whose average BMI was in the “normal” range. Our motivation for investigating motor variability in gait, however, is to investigate stride-to-stride control of older adults as it relates to fall risk [3]. Confirmation of validity and sensitivity of an IMU-driven biomechanical model is still needed in older populations living with and without neurological conditions. 

To mitigate differences attributed to different initial model poses in our evaluation of validity, we decided to offset the optoelectronic model to match the standing pose of the IMU model at the first frame, similar to the approach of Al Borno et al. [15]. This is unrealistic for situations where an optoelectronic system is unavailable to establish the initial IMU model pose. Adding the average correction (3.8° [15]) to the RMSD differences would produce differences that exceed limits tolerable for clinical applications [29]. However, ROM and motor variability were unaffected by this offset, and further IMU calibration procedures [58] are unlikely to affect validity or sensitivity of these measures. Thus, we expect that our conclusions on the validity of our outcomes can be generalized to situations where only IMU sensors are available.

Finally, we investigated performance of the IMU-driven model during treadmill gait, where stride-to-stride variability is lower than in overground gait [59,60]. Treadmills allow for analyses at constant speed and over a large number of continuous strides; for example, more than 100 strides are needed to reliably quantify SD of stride time and λ_max_ of tridimensional trunk acceleration [40]. Large numbers of strides can be recorded in optoelectronic-based overground studies when gait involves turning and strides are discontinuous [61] but calculation of local dynamic stability requires continuous time series. Use of a treadmill was the only practical way for us to quantify motor variability of straight-line continuous walking with the metrics we calculated and using the optoelectronic and IMU motion capture systems simultaneously. Simultaneous measurement with these systems was necessary to test the validity of our model. We are unaware of a clear reason that our findings would not hold for overground gait since our IMU orientations do not rely on magnetometers, which are sensitive to fluctuations in heterogeneity of the local magnetic field, and the suitability of IMU-driven biomechanical models for joint kinematic evaluations in overground locomotion is promising [14,15,27]. Nonetheless, the sensitivity of the IMU-driven model should be confirmed for overground evaluations of stride-to-stride control.

## 5. Conclusions

In summary, excluding pelvis tilt and swing-phase hip rotation and ankle inversion, IMU-based joint angle time series were acceptably accurate from the trunk down, ROM was acceptably consistent and accurate in the primary plane of motion, and magnitude of variability and local dynamic stability were acceptably consistent and accurate in all planes of motion. Validity was supported by the sensitivity of the IMU model to gait speed and arm swing amplitude-related responses in ROM, magnitude of variability, and local dynamic stability in the majority of cases. However, IMU-modelled estimates of ROM fluctuation persistence and of angle regularity were not acceptably consistent or accurate. We conclude that, for moderate-duration walking at slow and fast speeds, the IMU-driven, magnetometer-free, open-source biomechanical model used in this study provides valid estimates of joint angle time series, ROM in the primary plane of motion, stride-to-stride magnitude of variability, and stride-to-stride local dynamic stability which are also sensitive to within-participant responses. This provides a new way to evaluate biomechanical control of walking outside of the lab and clinic.

## Figures and Tables

**Table 1:** Root mean squared differences (RMSD) and RMSD relative to optoelectronic-modelled range of motion (CV_rom_) of the IMU-modelled joint angle time series during gait (N = 200 strides). Values are group means [95% confidence intervals]. Highlights represent pooled differences accepted as reasonable (green: RMSD ≤ 5.0°), differences approaching reasonable levels (yellow: 5.0° < RMSD ≤ 7.0°), and differences exceeding reasonable levels (orange: RMSD > 7.0°).

	Angle	Gait Condition					
		Preferred Speed, Preferred Swing	70% Preferred Speed, Preferred Swing	130% Preferred Speed, Preferred Swing	Preferred Speed, Active Swing	Preferred Speed, Arms Bound	Pooled
**RMSD (** **°** **)**	Trunk FE	3.8 [2.1, 5.5]	4.1 [2.1, 5.5]	4.2 [2.2, 6.2]	4.2 [2.8, 5.6]	3.4 [2.0, 4.8]	4.0 [2.3, 5.6]
	Trunk AA	3.6 [2.8, 4.3]	3.6 [2.7, 4.5]	3.9 [3.1, 4.7]	4.3 [3.3, 5.2]	3.4 [2.8, 4.0]	3.7 [2.9, 4.5]
	Trunk IE	4.0 [3.1, 5.0]	2.9 [2.5, 3.3]	3.8 [3.2, 4.4]	6.7 [1.9, 11.4]	2.9 [2.5, 3.4]	4.1 [2.6, 5.5]
	Pelvis FE	10.6 [7.7, 13.5]	9.6 [6.7, 12.5]	8.1 [5.2, 10.9]	9.2 [6.1, 12.2]	8.8 [5.4, 12.2]	9.2 [6.2, 12.3]
	Pelvis AA	2.1 [1.7, 2.5]	2.3 [1.7, 2.8]	2.2 [1.9, 2.5]	2.4 [1.9, 2.5]	2.0 [1.6, 2.4]	2.2 [1.8–2.6]
	Pelvis IE	1.9 [1.5, 2.2]	1.7 [1.3, 2.2]	2.1 [1.7, 2.5]	2.0 [1.6, 2.4]	1.8 [1.5, 2.2]	1.9 [1.5, 2.3]
	Hip FE	4.4 [3.5, 5.2]	3.7 [3.0, 4.4]	5.8 [4.9, 6.8]	4.2 [3.4, 4.9]	4.5 [3.0, 6.0]	4.5 [3.6, 5.5]
	Hip AA	5.5 [4.6, 6.4]	4.8 [4.0, 5.6]	5.8 [4.9, 6.7]	5.4 [4.4, 6.4]	5.5 [4.6, 6.5]	5.4 [4.5, 6.3]
	Hip IE	6.1 [5.4, 6.8]	5.8 [4.9, 6.6]	6.5 [5.7, 7.2]	5.8 [5.0, 6.7]	6.7 [5.5, 7.9]	6.2 [5.3, 7.0]
	Knee FE	4.6 [3.7, 5.5]	3.7 [3.1, 4.3]	4.8 [3.6, 5.9]	4.0 [3.6, 4.4]	4.7 [3.7, 5.6]	4.3 [3.5, 5.1]
	Ankle FE	6.7 [5.6, 7.9]	6.2 [4.9, 7.5]	7.5 [6.2, 8.8]	6.9 [5.4, 8.4]	7.4 [6.0, 8.8]	6.9 [5.6, 8.3]
	Ankle AA	12.0 [10.3, 13.6]	10.2 [8.8, 11.5]	12.0 [10.7, 13.2]	11.5 [10.1, 12.9]	12.7 [10.9, 14.5]	11.7 [10.2, 13.2]
	Pooled (all)	5.4 [5.0, 5.9]	4.9 [4.5, 5.2]	5.6 [5.2, 5.9]	5.5 [5.0, 6.0]	5.3 [4.9, 5.8]	5.3 [4.9, 5.8]
	Pooled (without Ankle AA)	4.8 [4.4, 5.3]	4.4 [4.1, 4.7]	5.0 [4.6, 5.4]	5.0 [4.4, 5.6]	4.6 [4.2, 5.1]	4.8 [4.3, 5.2]
**CV_rom_ (%)**	Trunk FE	25.5 [16.6, 34.3]	27.9 [17.8, 38.1]	29.4 [16.9, 42.0]	27.7 [19.6, 35.8]	28.2 [17.5, 38.9]	27.7 [17.7, 37.8]
	Trunk AA	15.0 [13.0, 17.1]	16.8 [13.7, 20.0]	15.3 [13.0, 17.7]	17.7 [14.5, 20.8]	17.9 [15.1, 19.2]	16.6 [13.9, 19.2]
	Trunk IE	15.6 [14.2, 37.8]	14.2 [12.0, 16.5]	13.7 [11.3, 16.1]	22.1 [3.8, 40.4]	15.6 [13.7, 17.5]	16.2 [10.5, 21.9]
	Pelvis FE	120.3 [80.3, 160.3]	94.2 [64.7, 123.7]	95.3 [55.5, 135.0]	92.9 [59.5, 126.2]	107.3 [58.5, 156.0]	102.0 [63.7, 140.2]
	Pelvis AA	12.3 [10.5, 14.1]	15.2 [11.0, 19.4]	12.0 [10.5, 13.5]	13.4 [11.8, 15.1]	14.4 [11.2, 17.7]	13.5 [11.0, 16.0]
	Pelvis IE	13.4 [10.4, 16.3]	12.9 [9.2, 16.5]	12.8 [10.7, 14.9]	11.7 [9.9, 13.6]	13.2 [10.8, 15.7]	12.8 [10.2, 15.4]
	Hip FE	9.1 [7.5, 10.6]	8.5 [6.6, 10.5]	11.0 [9.3, 12.6]	8.0 [6.6, 9.4]	9.2 [6.7, 11.8]	9.2 [7.3, 11.0]
	Hip AA	23.3 [18.5, 28.2]	21.9 [17.2, 26.5]	22.9 [18.8, 27.0]	22.1 [17.3, 27.0]	28.2 [21.5, 35.0]	23.7 [18.7, 28.7]
	Hip IE	32.2 [27.5, 37.0]	30.2 [25.0, 36.1]	32.9 [29.6, 36.1]	29.8 [25.4, 34.1]	35.0 [29.2, 40.9]	32.0 [27.3, 36.7]
	Knee FE	6.7 [5.4, 8.0]	5.5 [4.6, 6.4]	7.0 [5.5, 8.5]	6.0 [5.4, 6.7]	6.8 [5.3, 8.3]	6.4 [5.2, 7.6]
	Ankle FE	16.2 [13.9, 18.5]	17.2 [14.3, 20.2]	17.7 [15.2, 20.2]	16.6 [13.3, 19.9]	17.6 [14.5, 20.6]	17.1 [14.3, 19.9]
	Ankle AA	46.1 [39.6, 52.5]	41.4 [35.4, 47.4]	46.1 [40.8, 51.3]	43.5 [37.3, 49.6]	49.8 [43.0, 56.5]	45.3 [39.2, 51.5]
	Pooled (all)	28.0 [23.9, 32.1]	25.5 [22.5, 28.5]	26.3 [22.7, 30.0]	26.0 [22.2, 29.7]	28.6 [24.2, 33.0]	26.9 [23.1, 30.7]
	Pooled (without Ankle AA)	26.3 [22.2, 30.5]	24.1 [21.0, 27.1]	24.5 [20.7, 28.4]	24.4 [20.5, 28.3]	26.7 [21.9, 31.5]	25.2 [21.2, 29.2]

FE: flexion/extension; AA: abduction/adduction; IE: internal/external rotation.

**Table 2:** Validity of IMU-modelled vs. optoelectronic-modelled joint angle outcomes for gait. Outcomes are range of motion (ROM), mean standard deviation (meanSD), local divergence exponent (λ_max_), detrended fluctuation analysis scaling exponent for range of motion (DFAα), and sample entropy (SaEn). Validity metrics are intraclass correlation coefficients (ICC_2,1_), root mean square difference (RMSD), coefficient of variation of the optoelectronic mean (CV_mean_), IMU–optoelectronic bias, and 95% limits of agreement (LOA_95%_). Values are group means [95% confidence intervals]. Highlights represent excellent (dark green: ICC_2,1_ ≥ 0.75), good (green: 0.60 ≤ ICC_2,1_ < 0.75), fair (yellow: 0.40 ≤ ICC_2,1_ < 0.60), and poor (orange: ICC_2,1_ < 0.40) consistency.

	Angle	ICC_2,1_	RMSD	CV_mean_	Bias	LOA_95%_	
						Lower	Upper
**ROM**	Trunk FE	0.13 [−0.11, 0.35]	2.5 [2.1, 2.9]	26.1 [22.3, 29.8]	−1.3 [−1.8, −0.8] *	−5.4 [−5.9, −4.9]	2.8 [2.3, 3.3]
	Trunk AA	0.48 [0.28, 0.64]	7.8 [7.0, 8.6]	41.3 [38.3, 44.4]	−7.0 [−7.8, −6.2] *	−13.6 [−14.3, −12.8]	−0.5 [−1.3, 0.3]
	Trunk IE	0.85 [0.77, 0.90]	7.7 [6.8, 8.6]	38.3 [34.8, 41.7]	−6.7 [−7.6, −5.8] *	−14.1 [−15.0, −13.2]	0.7 [−0.2, 1.6]
	Pelvis FE	0.03 [−0.26, 0.21]	1.8 [1.5, 2.1]	26.2 [20.8, 31.5]	−0.4 [−0.8, 0.0] *	−3.8 [−4.2, −3.4]	3.0 [2.6, 3.4]
	Pelvis AA	0.62 [0.46, 0.75]	4.8 [4.2, 5.3]	32.6 [28.5, 35.6]	−4.1 [−4.7, −3.6] *	−8.8 [−9.4, −8.2]	0.5 [0.0, 1.1]
	Pelvis IE	0.84 [0.75, 0.90]	2.0 [1.7, 2.4]	14.8 [12.1, 17.6]	−0.5 [−0.9, 0.0] *	−4.4 [−4.9, −3.9]	3.5 [3.0, 4.0]
	Hip FE	0.84 [0.75, 0.90]	2.8 [2.4, 3.3]	5.0 [3.8, 6.1]	0.1 [−0.6, 0.8]	−5.5 [−6.2, −4.8]	5.7 [5.0, 6.4]
	Hip AA	0.24 [−0.01, 0.45]	8.5 [7.5, 9.6]	44.8 [36.9, 52.7]	6.8 [5.5, 8.0] *	−3.5 [−4.9, −2.3]	17.1 [15.8, 18.3]
	Hip IE	−0.11 [−0.35, −0.13]	4.8 [4.2, 5.4]	36.9 [30.4, 43.4]	0.4 [−0.7, 1.6]	−9.1 [−10.2, −7.9]	9.9 [8.7, 11.0]
	Knee FE	0.26 [0.02, 0.47]	6.9 [5.9, 7.9]	8.8 [7.2, 10.3]	−1.4 [−3.0, 0.2]	−14.7 [−16.4, 13.1]	11.9 [10.3, 13.5]
	Ankle FE	0.76 [0.64, 0.85]	4.7 [4.1, 5.4]	12.4 [10.3, 14.5]	−1.2 [−2.3, −0.1] *	−10.3 [−11.4, −9.2]	7.8 [6.7, 8.9]
	Ankle AA	0.10 [−0.14, 0.34]	8.4 [7.2, 9.6]	33.0 [26.6, 39.3]	3.1 [1.2, 5.0] *	−12.3 [−14.2, −10.4]	18.5 [16.6, 20.3]
**meanSD**	Trunk FE	0.63 [0.46, 0.75]	0.29 [0.25, 0.34]	17.8 [14.1, 21.6]	0.07 [0.00, 0.14] *	−0.49 [−0.56, −0.42]	0.63 [0.56, 0.70]
	Trunk AA	0.79 [0.68, 0.86]	0.18 [0.15, 0.22]	10.9 [8.6, 13.1]	0.02 [−0.02, 0.07]	−0.34 [−0.38, −0.29]	0.38 [0.34, 0.43]
	Trunk IE	0.80 [0.70, 0.87]	0.60 [0.54, 0.66]	36.3 [33.6, 39.1]	−0.52 [−0.59, −0.45] *	−1.10 [−1.17, −1.03]	0.06 [−0.01, 0.13]
	Pelvis FE	0.77 [0.65, 0.85]	0.16 [0.13, 0.18]	12.7 [10.1, 15.2]	0.04 [0.00, 0.07] *	−0.26 [−0.30, −0.22]	0.33 [0.30, 0.37]
	Pelvis AA	0.60 [0.43, 0.73]	0.14 [0.12, 0.17]	12.7 [10.2, 15.2]	−0.08 [−0.11, −0.05] *	−0.32 [−0.35, −0.29]	0.16 [0.13, 0.19]
	Pelvis IE	0.64 [0.48, 0.76]	0.28 [0.23, 0.32]	19.7 [15.1, 24.2]	0.20 [0.15, 0.24] *	−0.19 [−0.242, −0.15]	0.59 [0.54, 0.63]
	Hip FE	0.80 [0.69, 0.87]	0.23 [0.20, 0.26]	17.5 [14.4, 20.6]	0.16 [0.12, 0.20] *	−0.16 [−0.20, −0.12]	0.48 [0.44, 0.52]
	Hip AA	0.70 [0.56, 0.81]	0.14 [0.12, 0.16]	12.6 [10.1, 15.0]	0.06 [0.03, 0.09] *	−0.18 [−0.21, −0.15]	0.30 [0.27, 0.33]
	Hip IE	0.69 [0.54, 0.80]	0.20 [0.17, 0.23]	12.5 [9.9, 15.1]	0.10 [0.06, 0.14] *	−0.24 [−0.28, −0.20]	0.44 [0.40, 0.48]
	Knee FE	0.48 [0.27, 0.65]	0.40 [0.34, 0.46]	20.7 [15.6, 25.9]	0.19 [0.10, 0.27] *	−0.51 [−0.60, −0.43]	0.89 [0.80, 0.97]
	Ankle FE	0.68 [0.53, 0.79]	0.61 [0.55, 0.66]	50.0 [43.5, 56.5]	0.56 [0.50, 0.62] *	0.10 [−0.04, 0.16]	1.02 [0.97, 1.08]
	Ankle AA	0.24 [0.00, 0.46]	0.38 [0.32, 0.43]	25.6 [20.7, 30.5]	0.20 [0.13, 0.28] *	−0.42 [−0.49, −0.34]	0.83 [0.75, 0.90]
**λ_max_**	Trunk FE	0.72 [0.58, 0.82]	0.54 [0.48, 0.60]	14.3 [12.3, 16.2]	0.44 [0.37, 0.52] *	−0.18 [−0.25, −0.10]	1.06 [0.99, 1.14]
	Trunk AA	0.85 [0.77, 0.91]	0.40 [0.36, 0.44]	10.5 [9.3, 11.7]	0.36 [0.32, 0.40] *	0.04 [−0.00, 0.08]	0.69 [0.65, 0.72]
	Trunk IE	0.59 [0.40, 0.73]	0.34 [0.29, 0.39]	6.5 [5.3, 7.7]	−0.23 [−0.29, −0.17] *	−0.73 [−0.79, −0.67]	0.27 [0.21, 0.33]
	Pelvis FE	0.89 [0.82, 0.93]	0.61 [0.56, 0.66]	17.8 [16.1, 19.5]	0.58 [0.52, 0.63] *	0.16 [0.10, 0.21]	1.00 [0.95, 1.05]
	Pelvis AA	0.82 [0.71, 0.88]	0.37 [0.32, 0.41]	10.1 [8.6, 11.6]	0.32 [0.27, 0.36] *	−0.05 [−0.10, −0.01]	0.69 [0.64, 0.73]
	Pelvis IE	0.67 [0.51, 0.78]	0.22 [0.19, 0.25]	3.9 [3.1, 4.7]	−0.04 [−0.09, 0.01]	−0.47 [−0.52, −0.42]	0.39 [0.34, 0.44]
	Hip FE	0.87 [0.80, 0.92]	0.20 [0.17, 0.22]	4.2 [3.5, 4.9]	−0.04 [−0.09, 0.01]	−0.42 [−0.47, −0.37]	0.34 [0.29, 0.39]
	Hip AA	0.78 [0.66, 0.86]	0.31 [0.28, 0.34]	8.4 [7.5, 9.4]	0.27 [0.23, 0.30] *	−0.02 [−0.05, 0.02]	0.56 [0.52, 0.59]
	Hip IE	0.75 [0.62, 0.84]	0.44 [0.39, 0.49]	12.7 [10.9, 14.5]	0.38 [0.33, 0.43] *	−0.06 [−0.11, 0.00]	0.82 [0.77, 0.87]
	Knee FE	0.74 [0.61, 0.84]	0.40 [0.34, 0.46]	7.4 [5.9, 8.9]	0.20 [0.12, 0.28] *	−0.48 [−0.56, −0.40]	0.88 [0.79, 0.96]
	Ankle FE	0.74 [0.60, 0.83]	0.45 [0.41, 0.50]	13.0 [11.4, 14.5]	0.40 [0.35, 0.45] *	−0.02 [−0.07, 0.03]	0.82 [0.77, 0.87]
	Ankle AA	0.37 [0.14, 0.56]	1.17 [1.10, 1.25]	51.1 [45.9, 56.4]	1.13 [1.05, 1.21] *	0.49 [0.41, 0.56]	1.77 [1.69, 1.85]
**DFAα**	Trunk FE	0.40 [0.18, 0.58]	0.21 [0.18, 0.24]	22.7 [17.2, 28.2]	−0.03 [−0.08, 0.02]	−0.44 [−0.49, −0.39]	0.38 [0.33, 0.43]
	Trunk AA	0.27 [0.04, 0.48]	0.19 [0.16, 0.22]	18.7 [14.8, 22.6]	−0.05 [−0.09, 0.00]	−0.42 [−0.46, −0.37]	0.32 [0.28, 0.37]
	Trunk IE	0.65 [0.49, 0.76]	0.17 [0.15, 0.20]	16.6 [13.0, 20.2]	−0.00 [−0.04, 0.04]	−0.34 [−0.38, −0.30]	0.34 [0.30, 0.38]
	Pelvis FE	0.36 [0.14, 0.55]	0.20 [0.17, 0.23]	25.5 [20.4, 30.7]	0.01 [−0.04, 0.05]	−0.39 [−0.44, −0.25]	0.40 [0.35, 0.45]
	Pelvis AA	0.48 [0.28, 0.64]	0.21 [0.18, 0.24]	21.2 [17.7, 24.7]	−0.07 [−0.12, −0.03] *	−0.47 [−0.52, −0.42]	0.32 [0.27, 0.37]
	Pelvis IE	0.46 [0.25, 0.63]	0.16 [0.14, 0.19]	17.6 [13.3, 21.9]	0.03 [−0.01, 0.06]	−0.29 [−0.33, −0.25]	0.34 [0.30, 0.38]
	Hip FE	0.50 [0.30, 0.66]	0.20 [0.17, 0.23]	21.0 [16.8, 25.3]	−0.02 [−0.06, 0.03]	−0.41 [−0.46, −0.36]	0.38 [0.33, 0.43]
	Hip AA	0.37 [0.14, 0.56]	0.23 [0.20, 0.27]	21.3 [17.4, 25.1]	−0.07 [−0.12, −0.01] *	−0.50 [−0.56, −0.45]	0.37 [0.32, 0.43]
	Hip IE	0.13 [−0.12, 0.36]	0.24 [0.21, 0.27]	26.7 [21.8, 31.6]	−0.04 [−0.10, 0.02]	−0.51 [−0.56, −0.45]	0.43 [0.37, 0.48]
	Knee FE	0.43 [0.21, 0.61]	0.20 [0.17, 0.23]	20.0 [16.1, 23.9]	0.02 [−0.03, 0.07]	−0.37 [−0.42, −0.32]	0.41 [0.36, 0.46]
	Ankle FE	0.62 [0.44, 0.75]	0.16 [0.13, 0.18]	17.2 [13.4, 21.0]	0.02 [−0.02, 0.06]	−0.29 [−0.33, −0.25]	0.33 [0.29, 0.37]
	Ankle AA	−0.05 [−0.29, 0.20]	0.31 [0.35, 0.26]	35.9 [28.2, 43.7]	0.12 [0.05, 0.19] *	−0.44 [−0.51, −0.38]	0.68 [0.61, 0.75]
**SaEn**	Trunk FE	0.56 [0.38, 0.70]	0.18 [0.15, 0.20]	17.0 [13.8, 20.2]	0.02 [−0.02, 0.06]	−0.33 [−0.37, −0.29]	0.36 [0.32, 0.41]
	Trunk AA	0.61 [0.44, 0.74]	0.19 [0.16, 0.21]	29.8 [23.3, 36.3]	0.15 [0.13, 0.18] *	−0.05 [−0.08, −0.03]	0.36 [0.34, 0.39]
	Trunk IE	0.48 [0.28, 0.64]	0.10 [0.08, 0.12]	16.1 [12.3, 19.9]	0.04 [0.01, 0.06] *	−0.15 [−0.17, −0.13]	0.22 [0.20, 0.24]
	Pelvis FE	0.53 [0.34, 0.68]	0.22 [0.19, 0.26]	16.7 [14.1, 19.4]	−0.12 [−0.16, −0.07] *	−0.49 [−0.54, −0.45]	0.26 [0.22, 0.31]
	Pelvis AA	0.68 [0.54, 0.79]	0.12 [0.10, 0.13]	15.1 [12.1, 18.1]	0.06 [0.03, 0.08] *	−0.15 [−0.17, −0.12]	0.26 [0.23, 0.28]
	Pelvis IE	0.64 [0.48, 0.76]	0.24 [0.21, 0.27]	63.1 [47.9, 78.4]	0.19 [0.15, 0.22] *	−0.11 [−0.14, −0.07]	0.48 [0.45, 0.52]
	Hip FE	0.44 [0.23, 0.62]	0.05 [0.04, 0.06]	17.9 [14.3, 21.4]	0.04 [0.03, 0.04] *	−0.04 [−0.05, −0.03]	0.11 [0.10, 0.12]
	Hip AA	0.17 [−0.08, 0.39]	0.15 [0.13, 0.17]	23.5 [20.0, 27.0]	−0.09 [−0.12, −0.06] *	−0.33 [−0.36, −0.30]	0.15 [0.12, 0.18]
	Hip IE	−0.12 [−0.35, 0.12]	0.25 [0.22, 0.29]	21.3 [17.2, 25.4]	−0.04 [−0.10, −0.02] *	−0.53 [−0.59, −0.47]	0.46 [0.40, 0.52]
	Knee FE	0.74 [0.61, 0.83]	0.07 [0.05, 0.08]	16.5 [12.8, 20.2]	−0.02 [−0.04, −0.01] *	−0.14 [−0.16, −0.13]	0.10 [0.08, 0.11]
	Ankle FE	0.46 [0.24, 0.63]	0.13 [0.11, 0.14]	24.6 [20.2, 29.0]	0.10 [0.08, 0.12] *	−0.06 [−0.08, −0.04]	0.26 [0.24, 0.28]
	Ankle AA	0.10 [−0.14, 0.34]	0.19 [0.16, 0.22]	26.8 [22.1, 31.6]	−0.03 [−0.08, −0.01] *	−0.40 [−0.44, −0.35]	0.34 [0.29, 0.38]

FE: flexion/extension; AA: abduction/adduction; IE: internal/external rotation. * significant bias (95% confidence interval did not cross zero).

## Data Availability

The models underlying the findings of this study are freely available on SimTK (https://simtk.org/projects/bailey2021_imu (accessed on 2 November 2021)).

## Supplementary Materials

The following are available online at https://www.mdpi.com/article/10.3390/s21227690/s1, Figure S1: Inverse kinematics solutions to optoelectronic-based models with and without upper-limb weights, and to optoelectronic- and IMU-based models for 1- and 2-degree-of-freedom ankles, Figure S2: Removal of orientation drift from IMUs, Figure S3: Identification of heel strikes from optoelectronic- and IMU-modelled calcaneus linear velocities, Figure S4: Root-mean-squared difference of IMU-based joint angles relative to optoelectronic motion capture as a function of stride number, Figures S5–S9: Bland-Altman plots of joint angle range of motion and joint angle variability outcomes, Tables S1 and S2: Summarized optoelectronic- and IMU-based joint angle outcome data and sensitivity to gait speed and arm swing differences, Videos S1–S4: Animations of the optoelectronic-based and IMU-based biomechanical models for preferred speed and arm swing gait in a representative subject.
