# Supplementary material for: Validity and Sensitivity of an Inertial Measurement Unit-Driven Biomechanical Model of Motor Variability for Gait
Source: Sensors (Basel). 2021 Nov 19;21(22):7690. doi: 10.3390/s21227690 (PMC8626040; doi:10.3390/s21227690)
Supplement: Supplementary file 1 [file sensors-21-07690-s001.zip › Supplementary Figure S1.pdf]

**Supplementary Figure S1: Inverse kinematics solutions to optoelectronic-based models with and without upper-limb marker weights, and to optoelectronic- and IMU-based models for 1- and 2-degree-of-freedom ankles**

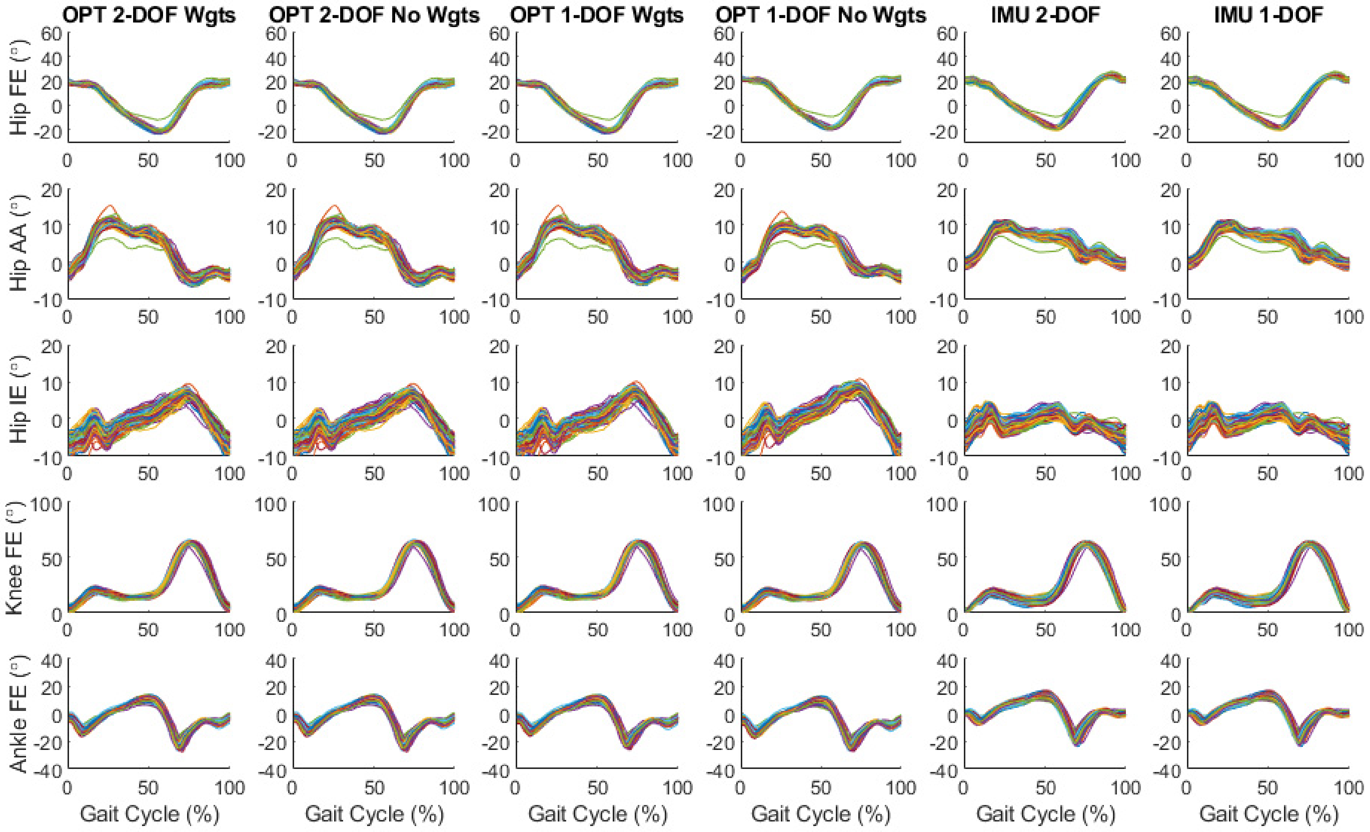


*Figure S1. Optoelectronic (OPT) and inertial measurement unit (IMU) modeled right lower-limb joint angles in 200 strides of preferred speed and arm swing gait in a representative participant. Hip flexion/extension (FE), hip abduction/adduction (AA), hip internal/external rotation (IE), knee FE, and ankle FE are compared over several models varying by ankle degrees of freedom (DOF) and weightings applied to upper-limb markers for calculating inverse kinematics. Columns from left to right: 1) an OPT-based 2-DOF ankle with equal upper- and lower-limb marker weights; 2) an OPT-based 2-DOF ankle with no upper-limb marker weights; 3) an OPT-based 1-DOF ankle with equal upper- and lower-limb marker weights; 4) an OPT-based 1-DOF ankle with no upper-limb marker weights; 5) an IMU-based 2-DOF ankle; 6) an IMU-based 1-DOF ankle. The 2-DOF ankle allows both FE and AA while the 1-DOF ankle allows only FE.*
