# Supplementary material for: Validity and Sensitivity of an Inertial Measurement Unit-Driven Biomechanical Model of Motor Variability for Gait
Source: Sensors (Basel). 2021 Nov 19;21(22):7690. doi: 10.3390/s21227690 (PMC8626040; doi:10.3390/s21227690)
Supplement: Supplementary file 1 [file sensors-21-07690-s001.zip › Supplementary Figure S2.pdf]

Supplementary Figure S2: Removal of orientation drift from IMUs

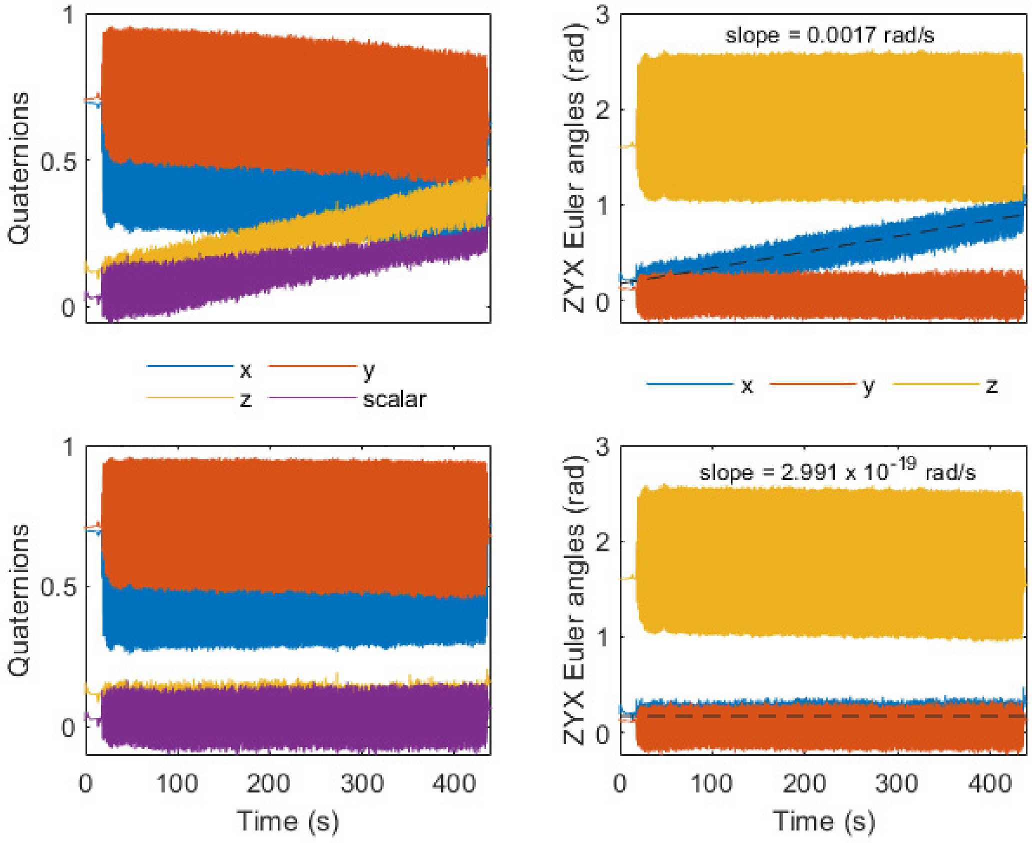

Figure S2. Orientation of the inertial measurement unit (IMU) on the left shank during a trial of gait at preferred speed and with preferred arm swing in a representative participant. Quaternion (left) and ZYX Euler angle (right) orientation representations are shown before (top) and after (bottom) drift removal. A slope larger than 0.0010 rad/s (i.e., a drift) was identified in the sensor heading (rotation about X in the IMU frame; top-right panel) using a linear line of best fit (black dashed line) and was reset to 0 rad/s using the 'detrend' function in Matlab.
