# Supplementary material for: Validity and Sensitivity of an Inertial Measurement Unit-Driven Biomechanical Model of Motor Variability for Gait
Source: Sensors (Basel). 2021 Nov 19;21(22):7690. doi: 10.3390/s21227690 (PMC8626040; doi:10.3390/s21227690)
Supplement: Supplementary file 1 [file sensors-21-07690-s001.zip › Supplementary Figure S3.pdf]

Supplementary Figure S3: Identification of heel strikes from optoelectronic- and IMU-modelled calcaneus linear velocities

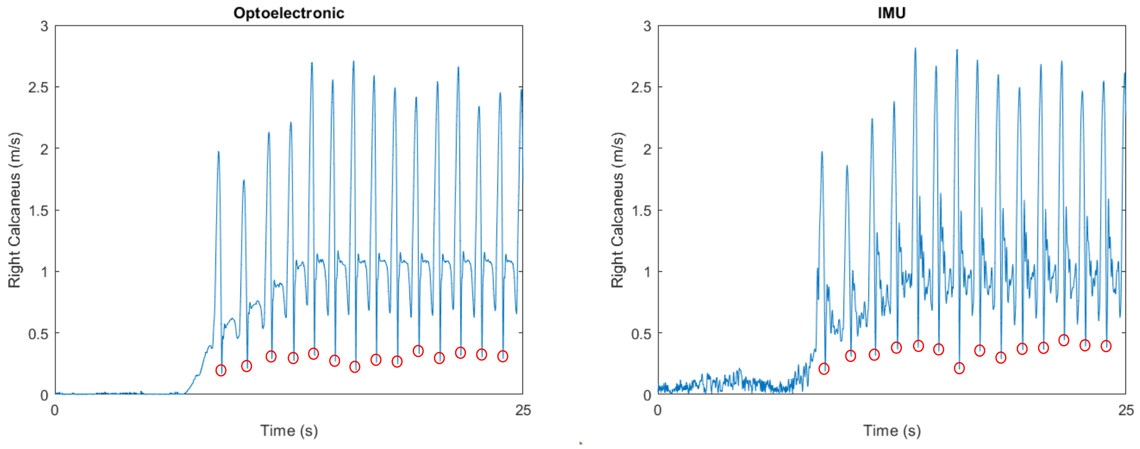

Figure S3. Filtered Euclidean mean linear velocity of the right calcaneus from the optoelectronic (left) and inertial measurement unit (IMU; right) models of preferred speed and arm swing gait for a representative participant. Heel strike events (red circles) were identified as the local minima immediately following each local maximum.
