# Supplementary material for: Validity and Sensitivity of an Inertial Measurement Unit-Driven Biomechanical Model of Motor Variability for Gait
Source: Sensors (Basel). 2021 Nov 19;21(22):7690. doi: 10.3390/s21227690 (PMC8626040; doi:10.3390/s21227690)
Supplement: Supplementary file 1 [file sensors-21-07690-s001.zip › Supplementary Figure S4.pdf]

Supplementary Figure S4: Root-mean-squared difference of IMU-based joint angles relative to optoelectronic motion capture as a function of stride number

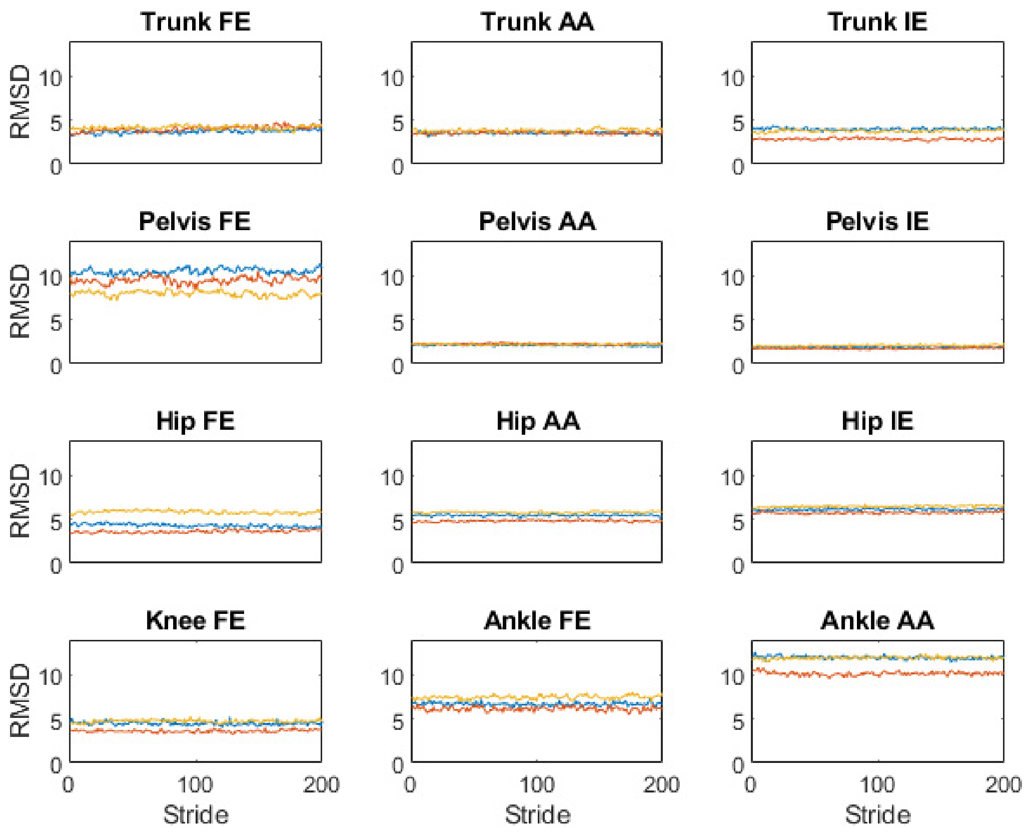

Figure S4. Mean root-mean-squared difference (RMSD) for each stride in the sagittal (FE), frontal (AA), and transverse (IE) planes for the trunk, pelvis, hip, knee, and ankle. RMSD is shown for the preferred speed (blue), 70% preferred speed (red), and 130% preferred speed (yellow) conditions.
