# Supplementary material for: Validity and Sensitivity of an Inertial Measurement Unit-Driven Biomechanical Model of Motor Variability for Gait
Source: Sensors (Basel). 2021 Nov 19;21(22):7690. doi: 10.3390/s21227690 (PMC8626040; doi:10.3390/s21227690)
Supplement: Supplementary file 1 [file sensors-21-07690-s001.zip › Supplementary Figures S5-S9.pdf]

Supplementary Figures S5–S9: Bland-Altman plots of joint angle range of motion and joint angle variability outcomes

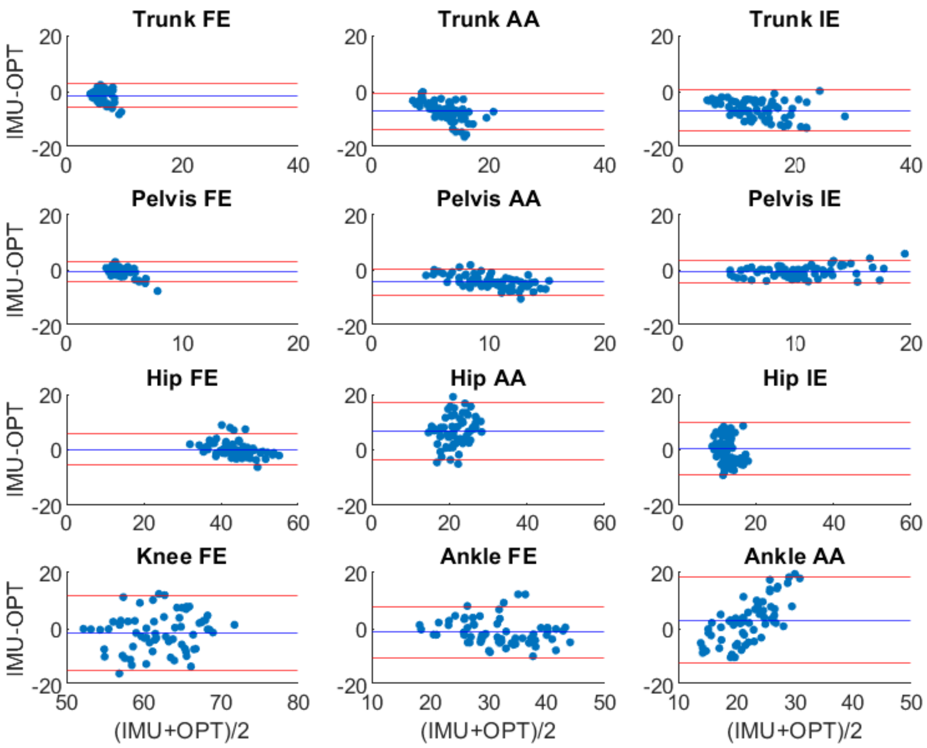

Figure S5. Bland-Altman plots of range of motion (ROM) of trunk, pelvis, and lower-limb joint angles for the optoelectronic-driven (OPT) and IMU-driven biomechanical models of constant-speed treadmill gait. Mean bias (blue line) and 95% limits of agreement (red lines) are shown. Values are in degrees (°). Angles include flexion/extension (FE), abduction/adduction (AA), and internal/external rotation (IE).

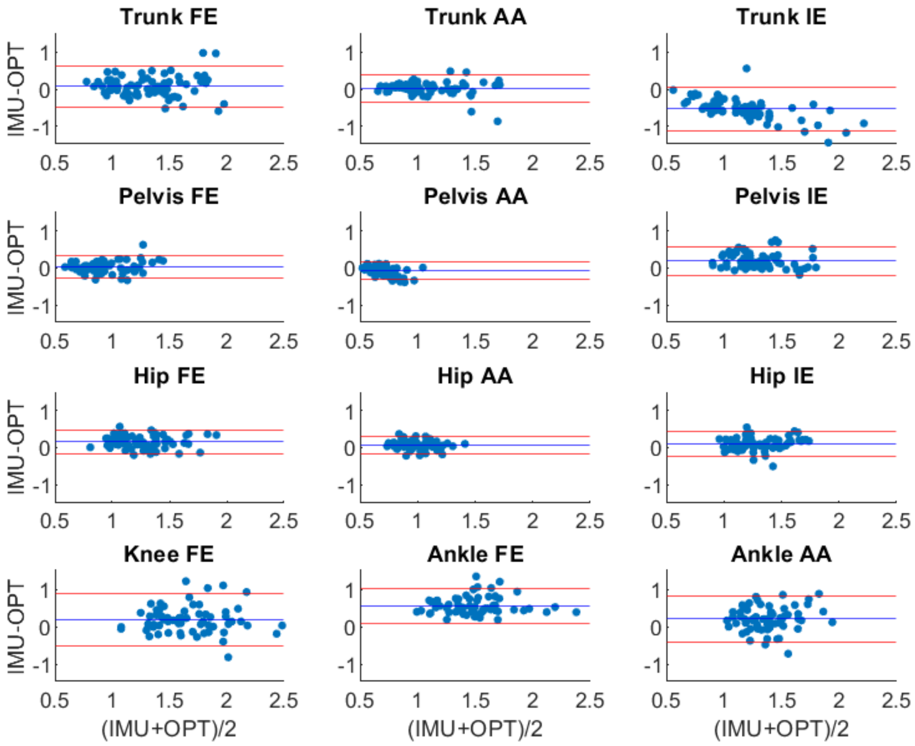

Figure S6. Bland-Altman plots of mean standard deviation (meanSD) of trunk, pelvis, and lower-limb joint angles for the optoelectronic-driven (OPT) and IMU-driven biomechanical models of constant-speed treadmill gait. Mean bias (blue line) and 95% limits of agreement (red lines) are shown. Values are in degrees (°). Angles include flexion/extension (FE), abduction/adduction (AA), and internal/external rotation (IE).

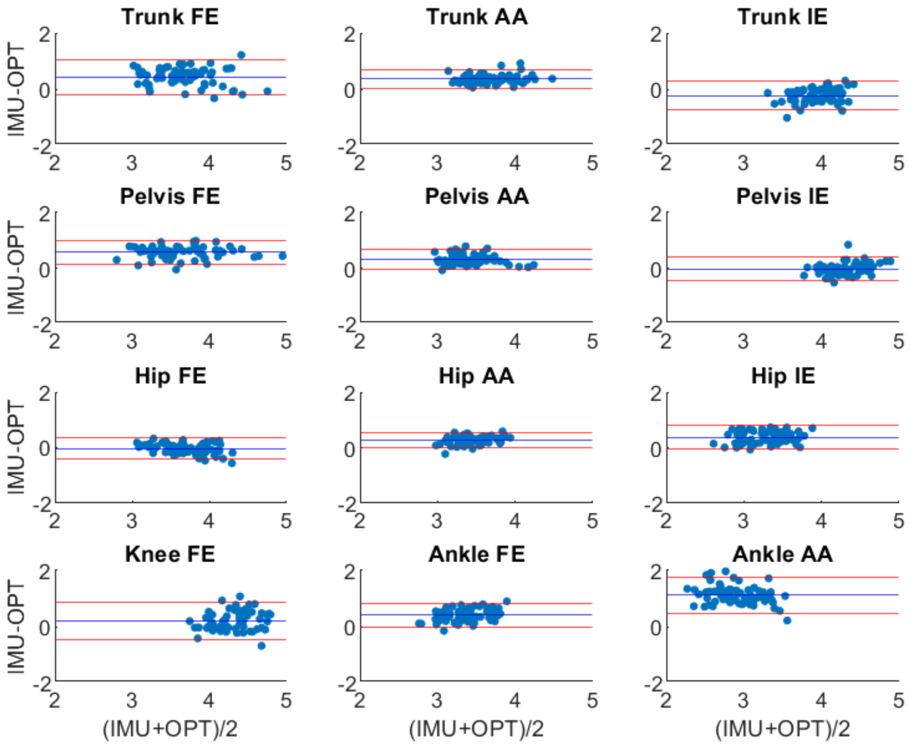

Figure S7. Bland-Altman plots of local divergence exponent (λ_{max}) of trunk, pelvis, and lower-limb joint angles for the optoelectronic-driven (OPT) and IMU-driven biomechanical models of constant-speed treadmill gait. Mean bias (blue line) and 95% limits of agreement (red lines) are shown. Values are in arbitrary units. Angles include flexion/extension (FE), abduction/adduction (AA), and internal/external rotation (IE).

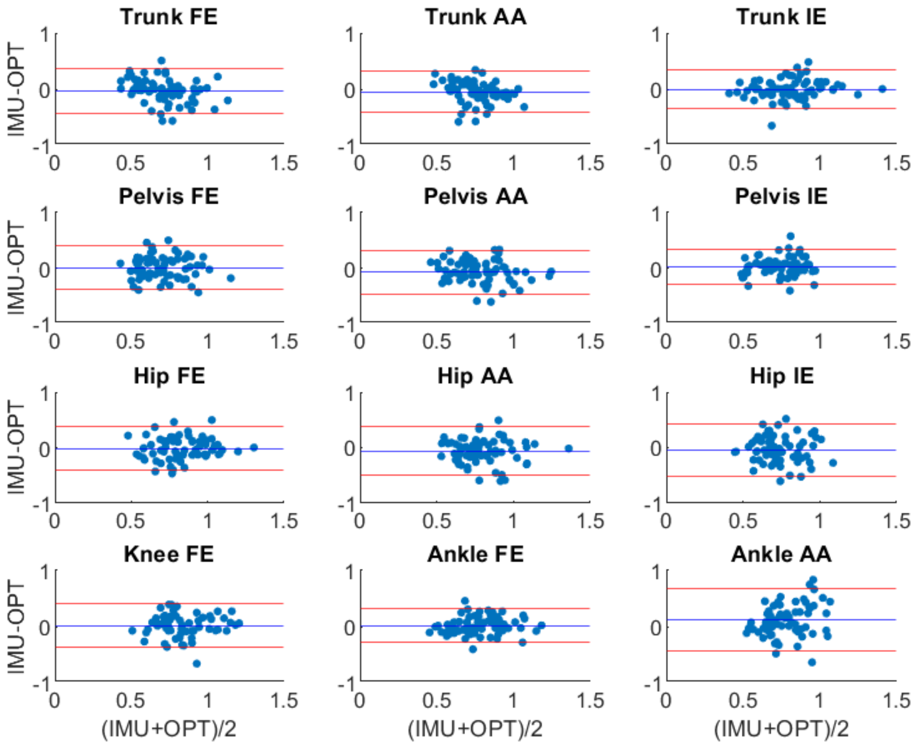

Figure S8. Bland-Altman plots of <u>detrended fluctuation analysis scaling exponent (DFAα)</u> of range of motion of trunk, pelvis, and lower-limb joint angles for the optoelectronic-driven (OPT) and IMU-driven biomechanical models of constant-speed treadmill gait. Mean bias (blue line) and 95% limits of agreement (red lines) are shown. Values are in arbitrary units. Angles include flexion/extension (FE), abduction/adduction (AA), and internal/external rotation (IE).

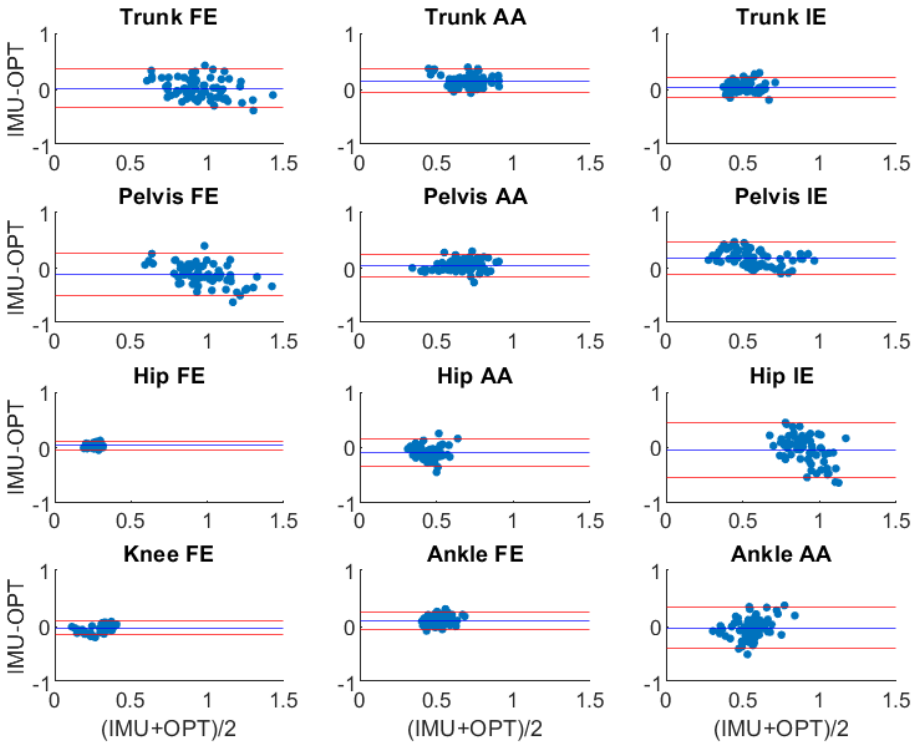

Figure S9. Bland-Altman plots of <u>sample entropy (SaEn)</u> of trunk, pelvis, and lower-limb joint angles for the optoelectronic-driven (OPT) and IMU-driven biomechanical models of constant-speed treadmill gait. Mean bias (blue line) and 95% limits of agreement (red lines) are shown. Values are in arbitrary units. Angles include flexion/extension (FE), abduction/adduction (AA), and internal/external rotation (IE).
