# Supplementary material for: Validity and Sensitivity of an Inertial Measurement Unit-Driven Biomechanical Model of Motor Variability for Gait
Source: Sensors (Basel). 2021 Nov 19;21(22):7690. doi: 10.3390/s21227690 (PMC8626040; doi:10.3390/s21227690)
Supplement: Supplementary file 1 [file sensors-21-07690-s001.zip › Supplementary Tables S1 and S2.pdf]

Supplementary Tables S1 and S2: Summarized optoelectronic- and IMU-based joint angle outcome data and sensitivity to gait speed and arm swing differences

Table S1. Means [95% confidence interval] for optoelectronic-based model outcomes across gait conditions. Outcomes are range of motion (ROM), mean standard deviation (meanSD), local divergence exponent (λ_{max}), detrended fluctuation analysis scaling exponent of range of motion (DFAα), and sample entropy (SaEn). P-values from repeated measures ANOVAs for effects of Speed and Swing are provided.

	**Angle**	**Gait condition**					**P-value**	
		Preferred speed, preferred swing	70% preferred speed, preferred swing	130% preferred speed, preferred swing	Preferred speed, active swing	Preferred speed, arms bound	Speed	Swing
**ROM**	Trunk FE [2]	6.5 [5.5,7.5]	6.5 [5.6,7.5]	6.4 [5.0,7.8]	7.3 [6.6,8.0]	5.9 [5.0-6.8]	0.91	0.003
	Trunk AA [a,b,2]	16.9 [14.8,19.0]	14.3 [11.5,17.1]	18.7 [16.8,20.6]	17.1 [14.9,19.2]	14.0 [11.9,16.1]	< 0.001	< 0.001
	Trunk IE [a,b,2]	17.4 [14.9,19.9]	13.9 [11.9,16.0]	20.4 [17.3,23.5]	22.2 [17.3,27.1]	12.7 [10.5,14.9]	< 0.001	< 0.001
	Pelvis FE	4.8 [3.8,5.7]	4.7 [3.9,5.4]	4.8 [3.4,6.1]	5.3 [4.6,5.9]	4.6 [3.6,5.5]	0.91	< 0.001
	Pelvis AA [a,b,2]	12.9 [11.0,14.7]	10.8 [8.4,13.2]	14.4 [12.8,16.1]	13.2 [11.5,14.9]	10.4 [8.7,12.1]	< 0.001	0.022
	Pelvis IE [a,b,1,2]	10.0 [8.7,11.4]	8.7 [7.4,10.1]	12.7 [10.8,14.6]	11.7 [9.8,13.5]	8.5 [6.5,10.5]	< 0.001	< 0.001
	Hip FE [a,b,1]	43.2 [40.6,45.8]	37.9 [35.7,40.1]	48.8 [46.1,51.4]	46.2 [43.6,48.8]	43.4 [40.6,46.2]	< 0.001	< 0.001
	Hip AA [b,2]	18.6 [16.6,20.7]	17.0 [14.2,19.7]	20.3 [18.7,21.9]	19.2 [17.1,21.4]	15.8 [14.117.5]	< 0.001	< 0.001
	Hip IE [a,b]	12.6 [10.5,14.8]	11.3 [9.25,13.2]	13.7 [11.3,16.2]	12.3 [9.8,14.8]	12.0 [10.0,13.9]	< 0.001	< 0.001
	Knee FE [a,1]	64.0 [61.1,67.0]	60.9 [57.7,64.1]	63.9 [60.7,67.1]	61.2 [58.0,64.3]	63.7 [60.7,66.7]	< 0.001	0.28
	Ankle FE [a,b]	33.5 [28.4,36.5]	27.3 [23.6,31.0]	35.3 [32.0,38.6]	32.3 [27.5,37.1]	33.6 [29.1,38.2]	< 0.001	< 0.001
	Ankle AA [a,b]	20.6 [18.6,22.5]	19.2 [17.7,20.6]	21.8 [19.6,23.9]	21.0 [19.0,23.0]	20.7 [19.0,22.4]	0.001	0.007
**meanSD**	Trunk FE	1.28 [1.07,1.49]	1.31 [1.13,1.49]	1.30 [1.12,1.48]	1.53 [1.29,1.77]	1.21 [1.03,1.39]	0.75	0.003
	Trunk AA [a,1,2]	1.05 [0.90,1.20]	1.18 [0.98,1.37]	1.05 [0.89,1.20]	1.34 [1.11,1.57]	0.87 [0.76,0.98]	0.002	< 0.001
	Trunk IE [1,2]	1.46 [1.29,1.62]	1.34 [1.20,1.48]	1.51 [1.32,1.69]	2.10 [1.84,2.37]	1.10 [0.96,1.24]	0.005	< 0.001
	Pelvis FE [a,1]	0.83 [0.68,0.99]	0.99 [0.87,1.11]	0.82 [0.71,0.94]	0.95 [0.84,1.06]	0.87 [0.74,0.99]	0.001	0.004
	Pelvis AA [a,1]	0.67 [0.57,0.78]	0.78 [0.67,0.88]	0.67 [0.58,0.76]	0.79 [0.69,0.88]	0.65 [0.55,0.74]	0.001	< 0.001
	Pelvis IE [b,1,2]	1.15 [0.99,1.31]	1.27 [1.13,1.41]	1.00 [0.88,1.13]	1.28 [1.11,1.46]	1.30 [1.16,1.45]	< 0.001	0.004
	Hip FE [a]	1.14 [0.94,1.33]	1.37 [1.22,1.51]	1.08 [0.95,1.20]	1.29 [1.15,1.42]	1.17 [1.03,1.31]	< 0.001	0.008
	Hip AA [a,1]	0.92 [0.81,1.03]	1.03 [0.92,1.14]	0.94 [0.84,1.04]	1.06 [0.96,1.16]	0.92 [0.83,1.02]	0.002	< 0.001
	Hip IE [b,1]	1.24 [1.10,1.38]	1.29 [1.16,1.41]	1.16 [1.05,1.27]	1.39 [1.28,1.50]	1.27 [1.11,1.43]	0.018	0.002
	Knee FE [a]	1.54 [1.26,1.81]	1.89 [1.67,2.11]	1.38 [1.23,1.53]	1.67 [1.51,1.83]	1.52 [1.36,1.68]	< 0.001	0.033
	Ankle FE [1]	1.21 [0.97,1.45]	1.30 [1.16,1.43]	1.12 [1.00,1.25]	1.39 [1.23,1.56]	1.22 [1.04,1.41]	0.026	0.001
	Ankle AA [a,b]	1.32 [1.12,1.52]	1.34 [1.21,1.48]	1.18 [1.07,1.29]	1.39 [1.27,1.51]	1.28 [1.14,1.43]	0.007	0.090
**λ_{max}**	Trunk FE [a,b,1]	3.37 [3.11,3.62]	3.78 [3.55,4.00]	3.16 [2.93,3.38]	3.66 [3.45,3.87]	3.33 [3.08,3.59]	< 0.001	< 0.001
	Trunk AA [a,1,2]	3.46 [3.33,3.59]	3.74 [3.57,3.91]	3.33 [3.20,3.45]	3.65 [3.52,3.75]	3.27 [3.11,3.44]	< 0.001	< 0.001
	Trunk IE [1,2]	4.06 [3.97,4.15]	4.12 [4.02,4.21]	4.01 [3.91,4.11]	4.34 [4.23,4.45]	3.80 [3.66,3.93]	0.007	< 0.001
	Pelvis FE [a,b,1]	3.21 [2.93,3.49]	3.72 [3.47,3.97]	3.02 [2.83,3.22]	3.50 [3.27,3.73]	3.25 [3.02,3.48]	< 0.001	< 0.001
	Pelvis AA [a,b,1]	3.21 [3.01,3.40]	3.54 [3.39,3.69]	3.08 [2.92,3.24]	3.37 [3.22,3.52]	3.12 [2.91,3.34]	< 0.001	< 0.001

	Pelvis IE [a,1,2]	4.15 [4.04,4.26]	4.37 [4.24,4.51]	4.16 [4.05,4.28]	4.50 [4.40,4.60]	4.41 [4.30,4.52]	0.001	< 0.001
	Hip FE [a,1]	3.60 [3.36,3.83]	3.97 [3.83,4.11]	3.48 [3.31,3.65]	3.80 [3.60,4.00]	3.67 [3.43,3.90]	< 0.001	0.006
	Hip AA [a,b,1]	3.24 [3.11,3.36]	3.53 [3.43,3.63]	3.14 [3.02,3.25]	3.42 [3.32,3.52]	3.28 [3.14,3.41]	< 0.001	< 0.001
	Hip IE [a,b,1,2]	2.99 [2.83,3.15]	3.33 [3.19,3.47]	2.76 [2.66,2.87]	3.24 [3.14,3.33]	3.08 [2.92,3.23]	< 0.001	0.002
	Knee FE [a,1]	4.11 [3.97,4.24]	4.59 [4.49,4.70]	3.95 [3.83,4.07]	4.24 [4.11,4.38]	4.10 [4.01,4.20]	< 0.001	0.004
	Ankle FE [a,1]	3.04 [2.92,3.16]	3.37 [3.26,3.48]	2.94 [2.83,3.05]	3.29 [3.15,3.43]	3.11 [2.99,3.22]	< 0.001	< 0.001
	Ankle AA [a,b]	2.36 [2.09,2.64]	2.71 [2.53,2.88]	2.02 [1.85,2.18]	2.42 [2.24,2.61]	2.22 [2.02,2.41]	< 0.001	0.068
**DFAα**	Trunk FE	0.68 [0.53,0.84]	0.68 [0.59,0.78]	0.69 [0.58,0.81]	0.83 [0.68,0.99]	0.68 [0.55,0.81]	0.96	0.12
	Trunk AA	0.74 [0.62,0.86]	0.78 [0.68,0.87]	0.71 [0.61,0.80]	0.82 [0.72,0.92]	0.89 [0.81,0.98]	0.58	0.26
	Trunk IE	0.77 [0.61,0.92]	0.81 [0.68,0.95]	0.80 [0.71,0.88]	0.79 [0.67,0.92]	0.83 [0.72,0.93]	0.81	0.61
	Pelvis FE	0.67 [0.54,0.79]	0.64 [0.55,0.72]	0.75 [0.66,0.84]	0.80 [0.67,0.92]	0.71 [0.60,0.83]	0.14	0.32
	Pelvis AA	0.86 [0.74,0.97]	0.86 [0.73,0.99]	0.74 [0.64,0.83]	0.84 [0.67,1.00]	0.85 [0.71,1.00]	0.13	0.90
	Pelvis IE	0.67 [0.59,0.75]	0.71 [0.62,0.80]	0.74 [0.65,0.83]	0.79 [0.70,0.88]	0.68 [0.60,0.76]	0.66	0.063
	Hip FE	0.78 [0.66,0.90]	0.82 [0.70,0.93]	0.84 [0.73,0.95]	0.92 [0.80,1.03]	0.84 [0.73,0.96]	0.76	0.15
	Hip AA	0.85 [0.72,0.99]	0.84 [0.69,0.98]	0.74 [0.65,0.82]	0.84 [0.73,0.96]	0.87 [0.73,1.00]	0.12	0.91
	Hip IE	0.71 [0.61,0.81]	0.77 [0.64,0.89]	0.69 [0.59,0.78]	0.83 [0.74,0.93]	0.79 [0.66,0.92]	0.49	0.21
	Knee FE	0.79 [0.65,0.93]	0.83 [0.72,0.94]	0.82 [0.72,0.92]	0.83 [0.73,0.94]	0.86 [0.76,0.97]	0.92	0.64
	Ankle FE	0.79 [0.67,0.92]	0.71 [0.59,0.82]	0.79 [0.72,0.85]	0.76 [0.68,0.85]	0.80 [0.68,0.92]	0.41	1.00
	Ankle AA	0.73 [0.66,0.81]	0.70 [0.62,0.79]	0.69 [0.57,0.80]	0.78 [0.66,0.90]	0.74 [0.62,0.85]	0.54	0.71
**SaEn**	Trunk FE [a,b,1]	0.96 [0.84,1.09]	0.83 [0.75,0.90]	1.09 [0.93,1.25]	0.85 [0.76,0.95]	0.98 [0.85,1.10]	< 0.001	< 0.001
	Trunk AA	0.62 [0.54,0.69]	0.60 [0.53,0.68]	0.64 [0.56,0.71]	0.62 [0.53,0.71]	0.66 [0.57,0.75]	0.399	0.12
	Trunk IE [a,b,2]	0.47 [0.42,0.51]	0.50 [0.45,0.55]	0.45 [0.40,0.50]	0.49 [0.43,0.56]	0.51 [0.47,0.55]	0.001	0.12
	Pelvis FE [a,1]	1.06 [0.90,1.21]	0.92 [0.83,1.01]	1.17 [0.99,1.34]	0.95 [0.82-1.08]	1.06 [0.92,1.21]	< 0.001	0.001
	Pelvis AA [2]	0.62 [0.55,0.69]	0.63 [0.55,0.72]	0.60 [0.54,0.67]	0.62 [0.55-0.69]	0.68 [0.59,0.77]	0.49	0.005
	Pelvis IE [b,1]	0.47 [0.35,0.59]	0.56 [0.46,0.66]	0.36 [0.25,0.46]	0.38 [0.28-0.49]	0.61 [0.48,0.74]	0.002	< 0.001
	Hip FE [2]	0.23 [0.21,0.25]	0.24 [0.22,0.25]	0.24 [0.22,0.26]	0.24 [0.22-0.25]	0.25 [0.22,0.27]	0.56	0.10
	Hip AA	0.48 [0.43,0.53]	0.47 [0.40,0.53]	0.50 [0.45,0.55]	0.48 [0.42-0.54]	0.52 [0.45,0.59]	0.24	0.025
	Hip IE [a]	0.94 [0.80,1.08]	0.82 [0.70,0.93]	1.00 [0.88,1.11]	0.94 [0.81-1.07]	0.93 [0.81,1.05]	< 0.001	0.95
	Knee FE	0.32 [0.28,0.36]	0.26 [0.20,0.31]	0.34 [0.32,0.37]	0.32 [0.28-0.36]	0.32 [0.29,0.35]	0.005	0.90
	Ankle FE [1]	0.45 [0.40,0.49]	0.46 [0.42,0.49]	0.46 [0.42,0.49]	0.49 [0.45-0.54]	0.43 [0.39,0.47]	0.67	< 0.001
	Ankle AA	0.47 [0.49,0.64]	0.53 [0.47,0.59]	0.58 [0.52,0.65]	0.59 [0.51-0.66]	0.57 [0.51,0.64]	0.054	0.65

FE: flexion/extension; AA: abduction/adduction; IE: internal/external rotation

[a] post-hoc difference between preferred speed and 70% preferred speed ($p < 0.05$)

[b] post-hoc difference between preferred speed and 130% preferred speed ($p < 0.05$)

[1] post-hoc difference between preferred swing and active swing ($p < 0.05$)

[2] post-hoc difference between preferred swing and bound swing ($p < 0.05$)

Table S2. Means [95% confidence interval] for IMU-based model outcomes across gait conditions. Outcomes are range of motion (ROM), mean standard deviation (meanSD), local divergence exponent (λ_{max}), detrended fluctuation analysis scaling exponent of range of motion (DFAα), and sample entropy (SaEn). P-values from repeated measures P-values from repeated measures ANOVAs for effects of Speed and Swing are provided.

	**Angle**	**Gait condition** Preferred speed, preferred swing	70% preferred speed, preferred swing	130% preferred speed, preferred swing	Preferred speed, active swing	Preferred speed, bound swing	**P-value** Speed	Swing
**ROM**	Trunk FE	5.4 [4.5,6.3]	6.0 [5.0,6.9]	5.3 [4.5,6.0]	5.3 [4.5,6.1]	4.7 [4.1,5.3]	0.014	0.02
	Trunk AA [a,b]	10.0 [9.1,11.0]	7.9 [6.9,9.0]	11.6 [10.2,13.1]	8.5 [7.7,9.3]	8.4 [7.7,9.0]	0.001	0.069
	Trunk IE [a,b,1,2]	10.1 [8.6,11.7]	7.8 [6.3,9.2]	12.7 [10.5,14.8]	15.5 [12.3,18.7]	6.4 [5.4,7.3]	0.002	< 0.001
	Pelvis FE	4.5 [4.1,4.8]	4.5 [4.1,5.0]	4.9 [4.5,5.3]	4.1 [3.6,4.5]	4.4 [4.1,4.7]	< 0.001	0.46
	Pelvis AA [a,b,2]	8.7 [7.6,9.7]	7.3 [6.2,8.5]	10.0 [8.7,11.2]	8.1 [7.0,9.2]	7.7 [6.7,8.6]	0.006	0.001
	Pelvis IE [b,1]	9.3 [7.4,11.2]	8.5 [6.7,10.2]	12.0 [9.2,14.8]	11.4 [9.3,13.6]	8.4 [6.1,10.7]	< 0.001	0.003
	Hip FE [a,b,1]	43.5 [41.1,45.9]	39.6 [37.5,41.6]	47.5 [45.3,49.7]	45.7 [43.3,48.2]	43.6 [41.3,45.9]	< 0.001	0.001
	Hip AA [a,b]	25.5 [22.6,28.4]	22.6 [19.7,25.4]	26.9 [24.0,29.8]	25.7 [22.8,28.6]	24.2 [21.8,26.5]	< 0.001	0.15
	Hip IE [a,b]	12.4 [11.1,13.8]	10.7 [9.9,11.4]	14.3 [12.8,15.7]	13.8 [12.1,15.4]	12.8 [10.8,14.9]	0.32	0.18
	Knee FE [1]	62.3 [58.9,65.8]	61.6 [57.3,65.9]	60.0 [56.7,63.2]	60.5 [56.9,64.0]	62.2 [58.5,66.0]	< 0.001	0.016
	Ankle FE [a,b]	31.5 [27.9,35.1]	26.9 [24.3,29.4]	34.3 [30.6,38.0]	30.6 [26.7,34.4]	31.6 [27.6,35.7]	0.083	0.19
	Ankle AA	22.9 [18.0,27.7]	20.0 [16.1,23.9]	24.4 [19.6,29.3]	24.9 [20.3,29.5]	26.4 [21.8,31.1]	0.73	0.32
**meanSD**	Trunk FE	1.34 [1.13,1.55]	1.40 [1.17,1.64]	1.39 [1.18,1.59]	1.46 [1.23,1.69]	1.34 [1.09,1.58]	0.72	0.02
	Trunk AA [1]	1.10 [0.95,1.25]	1.14 [0.96,1.33]	1.13 [0.97,1.30]	1.22 [1.09,1.35]	0.96 [0.79,1.12]	0.55	0.001
	Trunk IE	0.86 [0.75,0.97]	0.81 [0.69,0.93]	0.93 [0.78,1.08]	1.26 [1.09,1.43]	0.71 [0.62,0.79]	0.03	0.13
	Pelvis FE	0.91 [0.74,1.09]	1.06 [0.87,1.24]	0.83 [0.73,0.94]	0.98 [0.87,1.09]	0.92 [0.75,1.09]	0.008	0.12
	Pelvis AA [1]	0.61 [0.56,0.66]	0.64 [0.58,0.71]	0.62 [0.56,0.68]	0.67 [0.63,0.71]	0.58 [0.53,0.64]	0.33	0.003
	Pelvis IE [1]	1.33 [1.23,1.43]	1.40 [1.29,1.52]	1.31 [1.22,1.40]	1.61 [1.48,1.73]	1.40 [1.29,1.51]	0.028	< 0.001
	Hip FE	1.30 [1.11,1.49]	1.43 [1.29,1.57]	1.23 [1.09,1.37]	1.52 [1.35,1.68]	1.33 [1.18,1.48]	0.022	0.12
	Hip AA [1]	1.01 [0.92,1.09]	1.01 [0.92,1.09]	0.99 [0.89,1.08]	1.16 [1.07,1.25]	1.02 [0.92,1.11]	0.90	0.23
	Hip IE [a,1]	1.30 [1.12,1.48]	1.39 [1.26,1.52]	1.26 [1.15,1.37]	1.60 [1.48,1.71]	1.33 [1.19,1.46]	0.002	0.62
	Knee FE	1.69 [1.43,1.95]	1.92 [1.68,2.16]	1.52 [1.39,1.66]	1.89 [1.65,2.13]	1.78 [1.59,1.97]	0.004	0.28
	Ankle FE	1.77 [1.55,1.99]	1.86 [1.72,2.00]	1.67 [1.55,1.78]	2.02 [1.88,2.16]	1.78 [1.60,1.96]	0.036	0.042
	Ankle AA [1]	1.44 [1.24,1.63]	1.54 [1.35,1.74]	1.38 [1.26,1.50]	1.64 [1.43,1.85]	1.42 [1.27,1.56]	0.001	0.003
**λ_{max}**	Trunk FE [a,b,1]	3.81 [3.62,4.00]	4.27 [4.04,4.49]	3.50 [3.34,3.66]	4.04 [3.86,4.21]	3.90 [3.69,4.11]	< 0.001	0.008
	Trunk AA [a,b]	3.82 [3.68,3.96]	4.18 [4.03,4.33]	3.58 [3.45,3.71]	3.93 [3.77,4.09]	3.73 [3.55,3.91]	< 0.001	0.016
	Trunk IE [a,b,2]	3.84 [3.72,3.97]	4.15 [4.03,4.27]	3.59 [3.43,3.74]	3.93 [3.75,4.11]	3.57 [3.53,3.82]	< 0.001	0.008
	Pelvis FE [a,b,1,2]	3.79 [3.56,4.03]	4.29 [4.07,4.52]	3.50 [3.31,3.70]	4.06 [3.82,4.31]	3.94 [3.71,4.17]	< 0.001	0.005
	Pelvis AA [a,b,1,2]	3.51 [3.36,3.67]	3.87 [3.77,3.98]	3.28 [3.14,3.43]	3.63 [3.49,3.77]	3.60 [3.48,3.73]	< 0.001	0.007
	Pelvis IE [a,b,1,2]	4.13 [4.00,4.27]	4.55 [4.42,4.68]	4.00 [3.87,4.13]	4.41 [4.28,4.53]	4.30 [4.13,4.48]	< 0.001	< 0.001
	Hip FE [a,b]	3.66 [3.49,3.84]	4.08 [3.96,4.21]	3.42 [3.30,3.53]	3.77 [3.61,3.92]	3.70 [3.54,3.85]	< 0.001	0.17
	Hip AA [a,b,1]	3.49 [3.37,3.61]	3.82 [3.74,3.91]	3.28 [3.15,3.40]	3.64 [3.50,3.78]	3.53 [3.38,3.67]	< 0.001	0.31
	Hip IE [a,b,1]	3.39 [3.23,3.55]	3.73 [3.53,3.83]	3.18 [3.02,3.34]	3.61 [3.51,3.72]	3.38 [3.21,3.55]	< 0.001	0.55
	Knee FE [a]	4.32 [4.11,4.53]	4.67 [4.50,4.84]	4.01 [3.87,4.14]	4.29 [4.10,4.48]	4.24 [4.12,4.37]	< 0.001	0.28
	Ankle FE [a,b,1]	3.49 [3.37,3.62]	3.89 [3.78,3.99]	3.28 [3.15,3.41]	3.69 [3.55,3.82]	3.49 [3.38,3.60]	< 0.001	0.001

	Ankle AA [a,b]	3.44 [3.32,3.56]	3.77 [3.65,3.89]	3.19 [3.07,3.32]	3.61 [3.49,3.74]	3.48 [3.33,3.64]	< 0.001	0.034
**DFAα**	Trunk FE	0.69 [0.61,0.77]	0.70 [0.59,0.81]	0.65 [0.58,0.72]	0.67 [0.56,0.78]	0.69 [0.60,0.78]	0.72	0.94
	Trunk AA [2]	0.67 [0.61,0.73]	0.74 [0.67,0.81]	0.73 [0.64,0.83]	0.70 [0.61,0.79]	0.85 [0.79,0.92]	0.20	0.003
	Trunk IE	0.78 [0.63,0.93]	0.78 [0.64,0.93]	0.82 [0.71,0.92]	0.83 [0.67,1.00]	0.79 [0.67,0.90]	0.59	0.61
	Pelvis FE	0.69 [0.58,0.80]	0.66 [0.53,0.79]	0.71 [0.63,0.79]	0.75 [0.64,0.85]	0.74 [0.63,0.84]	0.90	0.54
	Pelvis AA	0.80 [0.69,0.91]	0.84 [0.76,0.92]	0.67 [0.57,0.78]	0.77 [0.65,0.89]	0.72 [0.61,0.83]	0.046	0.45
	Pelvis IE	0.73 [0.59,0.86]	0.73 [0.63,0.83]	0.75 [0.65,0.85]	0.81 [0.74,0.87]	0.75 [0.65,0.84]	0.95	0.47
	Hip FE	0.86 [0.72,1.00]	0.71 [0.59,0.83]	0.84 [0.72,0.95]	0.88 [0.76,1.00]	0.82 [0.69,0.96]	0.11	0.60
	Hip AA	0.77 [0.63,0.92]	0.77 [0.66,0.88]	0.69 [0.57,0.81]	0.85 [0.72,0.97]	0.73 [0.67,0.80]	0.28	0.37
	Hip IE	0.67 [0.56,0.78]	0.70 [0.60,0.81]	0.75 [0.61,0.88]	0.73 [0.65,0.81]	0.73 [0.63,0.84]	0.39	0.49
	Knee FE	0.82 [0.70,0.94]	0.91 [0.76,1.06]	0.86 [0.76,0.96]	0.81 [0.73,0.89]	0.83 [0.71,0.96]	0.68	0.83
	Ankle FE	0.80 [0.66,0.94]	0.77 [0.66,0.88]	0.79 [0.69,0.89]	0.79 [0.70,0.89]	0.79 [0.65,0.93]	0.74	0.91
	Ankle AA	0.84 [0.69,0.99]	0.85 [0.70,1.00]	0.97 [0.84,1.10]	0.82 [0.69,0.94]	0.75 [0.66,0.85]	0.20	0.74
**SaEn**	Trunk FE [a,b]	0.97 [0.90,1.05]	0.80 [0.74,0.86]	1.11 [1.00,1.21]	0.93 [0.85,1.01]	0.97 [0.88,1.06]	< 0.001	0.27
	Trunk AA	0.74 [0.68,0.80]	0.73 [0.67,0.80]	0.78 [0.71,0.86]	0.82 [0.75,0.89]	0.79 [0.73,0.84]	0.27	0.20
	Trunk IE [1,2]	0.51 [0.48,0.55]	0.54 [0.47,0.60]	0.49 [0.44,0.53]	0.44 [0.40,0.49]	0.62 [0.57,0.67]	0.09	< 0.001
	Pelvis FE [a,b]	0.93 [0.85,1.00]	0.80 [0.73,0.87]	1.00 [0.91,1.09]	0.92 [0.82,1.01]	0.88 [0.81,0.95]	< 0.001	0.50
	Pelvis AA	0.68 [0.60,0.76]	0.67 [0.58,0.77]	0.67 [0.59,0.74]	0.70 [0.61,0.78]	0.68 [0.59,0.76]	0.69	0.89
	Pelvis IE	0.71 [0.62,0.79]	0.64 [0.57,0.72]	0.65 [0.56,0.74]	0.64 [0.58,0.70]	0.71 [0.59,0.84]	0.12	0.20
	Hip FE	0.27 [0.25,0.30]	0.26 [0.23,0.28]	0.30 [0.28,0.31]	0.27 [0.24,0.29]	0.28 [0.25,0.30]	0.001	0.12
	Hip AA	0.37 [0.33,0.42]	0.39 [0.34,0.43]	0.42 [0.35,0.49]	0.40 [0.36,0.44]	0.41 [0.34,0.48]	0.076	0.36
	Hip IE	0.92 [0.85,0.97]	0.86 [0.79,0.92]	0.91 [0.85,0.96]	0.85 [0.79,0.90]	0.91 [0.81,1.01]	0.20	0.24
	Knee FE	0.28 [0.23,0.34]	0.22 [0.15,0.29]	0.35 [0.32,0.37]	0.31 [0.24,0.37]	0.30 [0.24,0.35]	< 0.001	0.11
	Ankle FE [1]	0.55 [0.49,0.60]	0.52 [0.48,0.55]	0.57 [0.52,0.62]	0.60 [0.54,0.65]	0.54 [0.49,0.59]	0.015	0.001
	Ankle AA	0.56 [0.46,0.66]	0.54 [0.47,0.62]	0.57 [0.46,0.68]	0.55 [0.43,0.67]	0.46 [0.38,0.54]	0.53	0.089

FE: flexion/extension; AA: abduction/adduction; IE: internal/external rotation

[a] post-hoc difference between preferred speed and 70% preferred speed ($p < 0.05$)

[b] post-hoc difference between preferred speed and 130% preferred speed ($p < 0.05$)

[1] post-hoc difference between preferred swing and active swing ($p < 0.05$)

[2] post-hoc difference between preferred swing and bound swing ($p < 0.05$)
