# Supplementary material for: Validity and Sensitivity of an Inertial Measurement Unit-Driven Biomechanical Model of Motor Variability for Gait
Source: Sensors (Basel). 2021 Nov 19;21(22):7690. doi: 10.3390/s21227690 (PMC8626040; doi:10.3390/s21227690)
Supplement: Supplementary file 1 [file sensors-21-07690-s001.zip › Supplementary Videos S1-S4.pdf]

Supplementary Videos S1–S4: Animations of the optoelectronic-based and IMU-based biomechanical models for preferred speed and arm swing gait in a representative subject

OPT_sagittal.webm

Video S1. Sagittal-plane view of the optoelectronic-based biomechanical model with spherical markers. Upper-limb bones and markers and lower-limb muscles have been hidden for visual clarity. Preferred speed and arm swing gait is shown for a representative participant.

OPT_front.webm

Video S2. Frontal-plane view of the optoelectronic-based biomechanical model with spherical markers. Upper-limb bones and markers and lower-limb muscles have been hidden for visual clarity. Preferred speed and arm swing gait is shown for a representative participant.

IMU_sagittal.webm

Video S3. Sagittal-plane view of the IMU-based biomechanical model with IMUs in the segment frame. Upper-limb bones and lower-limb muscles have been hidden for visual clarity. Preferred speed and arm swing gait is shown for a representative participant.

IMU_front.webm

Video S4. Frontal-plane view of the IMU-based biomechanical model with IMUs in the segment frame. Upper-limb bones and lower-limb muscles have been hidden for visual clarity. Preferred speed and arm swing gait is shown for a representative participant.
